# Lead‐Free Hybrid Perovskite Absorbers for Viable Application: Can We Eat the Cake and Have It too?

**DOI:** 10.1002/advs.201700331

**Published:** 2017-11-20

**Authors:** Lusheng Liang, Peng Gao

**Affiliations:** ^1^ CAS Key Laboratory of Design and Assembly of Functional Nanostructures and Fujian Provincial Key Laboratory of Nanomaterials Fujian Institute of Research on the Structure of Matter Chinese Academy of Sciences Fuzhou 350002 China; ^2^ Laboratory of Advanced Functional Materials Xiamen Institute of Rare Earth Materials Haixi Institute Chinese Academy of Sciences Xiamen 361021 China

**Keywords:** bismuth halide perovskite, lead‐free absorbers, low dimensional materials, perovskite solar cell, tin halide perovskite

## Abstract

Many years since the booming of research on perovskite solar cells (PSCs), the hybrid perovskite materials developed for photovoltaic application form three main categories since 2009: (i) high‐performance unstable lead‐containing perovskites, (ii) low‐performance lead‐free perovskites, and (iii) moderate performance and stable lead‐containing perovskites. The search for alternative materials to replace lead leads to the second group of perovskite materials. To date, a number of these compounds have been synthesized and applied in photovoltaic devices. Here, lead‐free hybrid light absorbers used in PV devices are focused and their recent developments in related solar cell applications are reviewed comprehensively. In the first part, group 14 metals (Sn and Ge)‐based perovskites are introduced with more emphasis on the optimization of Sn‐based PSCs. Then concerns on halide hybrids of group 15 metals (Bi and Sb) are raised, which are mainly perovskite derivatives. At the same time, transition metal Cu‐based perovskites are also referred. In the end, an outlook is given on the design strategy of lead‐free halide hybrid absorbers for photovoltaic applications. It is believed that this timely review can represent our unique view of the field and shed some light on the direction of development of such promising materials.

## 1 Introduction

**Figure 1:**
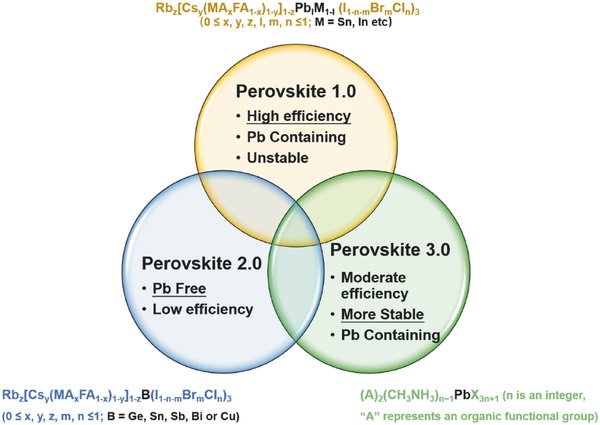
Classification of hybrid halide perovskites for photovoltaic application.

Owing to the increasing consumption of fossil energy and the deterioration of air pollution, there is an imperative need of clean and renewable energy resources for humanity. Among the new energy solutions, photovoltaic (PV) technology, which converts solar energy into electricity directly, is a promising approach to get sustainable clean energy safely. As one of the third‐generation PV technologies, hybrid halide perovskite solar cells (PSCs) emerged since Miyasaka and co‐workers^1^ incorporated MAPbX_3_ (X = I, Br) as sensitizers into dye‐sensitized solar cells (DSSCs), achieving a power conversion efficiency (PCE) of 3.8% in 2009. After numerous research endeavors in the past eight years, the PCEs of PSCs rapidly improved to 22.1%.^2^ Originally, hybrid halide perovskites with the general formula of ABX_3_ are structural analogs of the natural mineral CaTiO_3_, while A is a monovalent, organic or alkali metal cation, M is a divalent p‐block metal (typically Pb, Sn, and Ge), and X is a halide anion.^3^ Depending on the demand of the researchers, hybrid halide perovskites and their derivatives for the photovoltaic application can be classified into three main categories since 2009 (**Figure** **1**).

The first group of halide perovskites is dedicated to demonstrating the potential of PSCs in achieving PCE up to a theoretical upper limit of 31%.^4^ They are the most studied hybrid halide perovskites with the formula APb_l_M_1−l_X_3_ (A = CH_3_NH_3_, HC(NH_2_)_2_, Cs, Rb, or their mixture; M = Sn (II),^5^, ^6^, ^7^, ^8^ Ge (II),^9^, ^10^ Mn (II),^11^ Co (II),^12^ In (III),^13^ Al (III),^14^ or Sb (III),^15^ etc. or their mixture; X = Cl, Br, I, or their mixture) featuring the containing of Pb as the main metal cation and 3D network of corner‐sharing (Pb_1−l_M_l_)X_6_^4−^ octahedrons with the monovalent cation occupying the cuboctahedral cavity. The unique electronic configuration of Pb^2+^ endows 3D lead perovskites with excellent optoelectronic properties. For example, MAPbI_3_, the archetypal hybrid halide perovskite, possesses many ideal properties as a solar absorber: a direct bandgap (*E*_g_) of 1.53 eV,^16^ small exciton binding energies (37 or 45 meV),^17^, ^18^, ^19^ long charge carrier diffusion lengths over 3.5 µm,^20^, ^21^ and excellent charge carrier mobilities.^22^, ^23^, ^24^ Inherited all the merits of tribasic MAPbI_3_, the compositionally engineered polybasic 3D lead halide perovskite‐based PSCs exhibited a skyrocketing certified PCE from initial 14.9% to state‐of‐the‐art 22.1%^25^ within three years.

Aforementioned 3D lead halide perovskites realized the highest‐performing solution‐processed solar cell on record, rivaling commercial crystalline silicon solar cells in efficiency.^26^ However, the toxicity issue of the lead urged some researchers to seek alternatives to lead‐based perovskites. We classify these alternatives as the second group of perovskites, which features less toxic lead‐free hybrid halide light absorbers. Lead‐free hybrid halide light absorbers mainly include group 14 metals like tin (Sn) and germanium (Ge), group 15 posttransition metals like bismuth (Bi) and antimony (Sb), and transition metal copper (Cu) as the metal cations.^27^ In this case, a variety of crystallographic polymorphs appeared: Sn‐ and Ge‐based compounds with 3D perovskite framework; Bi‐ and Sb‐based “pseudoperovskite” without corner‐shared MX_6_ octahedra structure; Cu‐based typical 2D layered perovskites. As light absorbers used in solar cells, Sn‐based perovskites achieved the highest efficiency so far of 8.12% among all lead‐free hybrid halide compounds.^28^

In the group of high‐efficiency lead halide perovskites, there is another problem of insufficient long‐term stability for the application of the devices. One solution to this problem is mixing 3D perovskites with 2D perovskites,^29^, ^30^, ^31^, ^32^ which can be categorized as the third group of perovskites with the mission of realizing high efficiency and stability simultaneously. The mixed dimensional (MD) perovskites have a general chemical formula^30^ of (A)_2_(CH_3_NH_3_)*_n_*_−1_MX_3_ *_n_*_+1_ (*n* is an integer), where A is a primary aliphatic or aromatic alkylammonium cation, M is a divalent metal, and X is a halide anion. In the MD perovskites, the large organic cations (A) defragment the 3D structure and isolate certain number (*n*) of inorganic perovskite layers of corner‐sharing [MX_6_]^4−^ octahedrons.^33^ This configuration was found to prevent moisture from attacking the perovskite and therefore improve the stability of perovskite film. Additionally, the wide variety of “A and n” brings MD perovskites abundant tunability and flexibility to control the physical properties, as well as balanced stability versus optoelectronic performance of corresponding devices. So far, the quasi‐2D PEA_2_(CH_3_NH_3_)*_n_*_−1_Pb*_n_*I_3_ *_n_*_+1_ 32 and 2D perovskite (BA)_2_(MA)_3_Pb_4_I_13_ 34 displayed good efficiency over 12% through optimizing stoichiometry of materials and showed much improved stability than intrinsic 3D perovskites.

**Figure 2:**
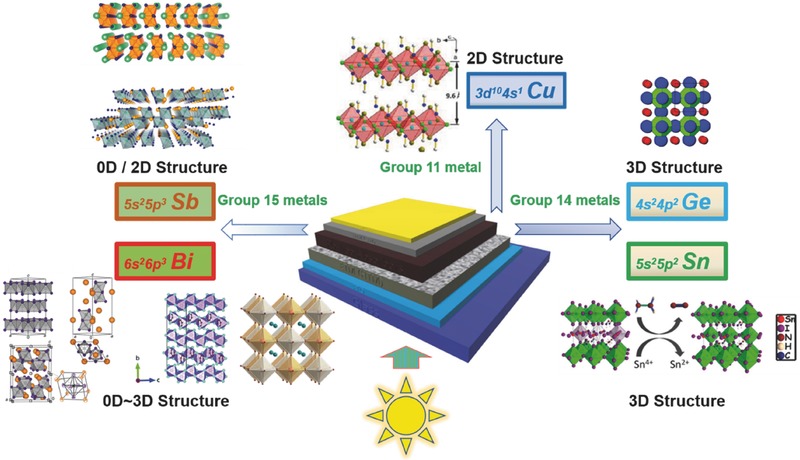
Scope of the lead‐free hybrid absorbers discussed in this review (Sb: Adapted with permission.^35^ Copyright 2016, ACS; Bi: Adapted with permission.^36^ Copyright 2015, ACS; Adapted with permission.^37^ Copyright 2017, RSC; Adapted with permission.^38^ Copyright 2016, ACS; Cu: Adapted with permission.^39^ Copyright 2016, ACS; Ge: Adapted with permission.^40^ Copyright 2015, RSC; Sn: Adapted with permission.^41^ Copyright 2017, ACS).

The three groups of hybrid perovskites attracted attention to different extents. In this series review, we focus on the second group of perovskites or lead‐free hybrid light absorbers used in photovoltaic devices. Recent development in structures, optoelectronic properties, and the related solar cell applications of these types of hybrid light absorbers are summarized. In the first part, group 14 metals (Sn and Ge)‐based perovskites are introduced with more emphasis on the optimization of Sn‐based PSCs. Then we put concerns on halide hybrids of group 15 metals (Bi and Sb), which are mainly perovskite derivatives. At the same time, transition metal Cu‐based perovskites are also referred. In the end, we give an outlook on the design strategy of lead‐free halide hybrid absorbers for photovoltaic applications (**Figure** **2**). An almost complete summary of the state‐of‐the‐art development of the lead‐free halide hybrid absorbers in PV devices is listed in **Table** **1**.

**Table 1:** Lead‐free hybrid absorbers comparing to the lead‐containing counterparts

Metal Cations		Absorber	PCE (%)	*V*_OC_ (V)	*J*_SC_ (mA cm^−2^)	FF (%)	*E*_g_ (eV)	Architecture	Fabrication method	Additive	Ref.
Pb		MAPbI_3_	20.4	1.11	23.7	77.3	1.55	FTO/c‐TiO_2_/mp‐TiO_2_/absorber/spiro‐OMeTAD/Au	Spin coating+ solvent‐engineering	No	42
		FAPbI_3_	18.1	1.04	23.2	74.9	1.45	FTO/c‐TiO_2_/mp‐TiO_2_/absorber/spiro‐OMeTAD/Au	Spin coating‐solvent bathing	No	43
		FA_1−_ *_x_*MA*_x_*PbI_3−_ *_y_*Br*_y_*	22.1	1.1	25.0	80.3	1.50	FTO/c‐TiO_2_/mp‐TiO_2_/absorber/PTAA/Au	Spin coating	I_2_	44
		Rb_0.05_[Cs_0.05_(MA_0.17_FA_0.83_)_0.95_]_0.95_Pb(I_0.83_Br_0.17_)_3_	21.8	1.18	22.8	81	1.63	FTO/c‐TiO_2_/mp‐TiO_2_/absorber/spiro‐OMeTAD/Au	Spin coating + solvent‐engineering	No	45
Sn(II)		MASnI_3_	6.4	0.88	16.8	42	1.2	FTO/c‐TiO_2_/mp‐TiO_2_/absorber/spiro‐OMeTAD/Au	Spin coating	No	46
		MASnI_3_	5.44	0.716	15.18	50.1	1.3	FTO/c‐TiO_2_/mp‐TiO_2_/absorber/spiro‐OMeTAD/Au	Spin coating	No	5
		MASnI_3_	3.89	0.38	19.9	51.7	1.3	FTO/c‐TiO_2_/mp‐TiO_2_/absorber/PTTA/Au	Spin coating (hydrazine vapor)	SnF_2_	41
		MASnI_3_	3.15	0.32	21.4	46	1.3	FTO/c‐TiO_2_/mp‐TiO_2_/absorber/Au	solvent‐engineering	SnF_2_	47
		MASnI_3_	2.14	0.45	11.8	40	–	ITO/PEDOT:PSS/absorber/C_60_/BCP/Ag	Spin coating‐solvent bathing	SnF_2_	48
		MASnI_3_	1.86	0.27	17.4	39.1	1.26	FTO/c‐TiO_2_/mp‐TiO_2_/absorber/PTTA/Au	VASP	No	49
		MASnI_3_	1.7	0.38	12.1	36.6	1.3	ITO/PEDOT:PSS/Poly‐TPD/absorber/ C_60_/BCP/Ag	thermal co‐evaporation	No	50
		MASnI_3_	–	–	–	–	1.23	–	vapor deposition + solution‐spinning		
		MASnI_3−_ *_x_*Br*_x_*	5.73	0.82	12.30	57	1.75	FTO/c‐TiO_2_/mp‐TiO_2_/absorber/spiro‐OMeTAD/Au	Spin coating	No	51
		MASnBr_3_ MA_0.9_Cs_0.1_SnI_3_	1.12	0.50	4.27	49.1	2.2	FTO/c‐TiO_2_/mp‐TiO_2_/absorber/P3HT/Au	sequential evaporation	No	52
			0.51	0.49	2.24	46.4	2.13	FTO/c‐TiO_2_/mp‐TiO_2_/absorber/PTTA/Au	(VASP)	No	53
			0.3	0.20	4.5	36	1.41	ITO/PEDOT:PSS/absorber/PCBM/Bis‐C_60_/Ag	Spin coating solvent‐engineering (Tol)	No	54
		{en}MASnI_3_	6.63	0.43	24.3	63.7	1.4	FTO/c‐TiO_2_/mp‐TiO_2_/absorber/PTTA/Au	Spin coating (hydrazine vapor)	SnF_2_	55
		FASnI_3_	6.6	0.48	21.3	64.6	1.36	ITO/PEDOT:PSS/absorber/C_60_/BCP/Ag	Spin coating solvent‐engineering (CB)	SnF_2_	28
		FASnI_3_	6.22	0.47	22.1	60.7	1.4	ITO/PEDOT:PSS/absorber/C_60_/BCP/Ag.	Spin coating solvent‐engineering (DEE)	SnF_2_	56
		FASnI_3_	5.27	0.38	23.1	60.0	1.4	FTO/c‐TiO_2_/mp‐TiO_2_/ZnS/absorber/ PTAA/Au	Spin coating solvent‐engineering (DEE)	SnF_2_	57
		FASnI_3_	4.8	0.32	23.7	63	1.4	FTO/c‐TiO_2_/mp‐TiO_2_/absorber/spiro‐OMeTAD/Au	Spin coating	SnF_2_‐pyrazine	58
		FASnI_3_	2.45	0.31	17.17	46.0	1.4	–	–	No	
		FASnI_3_	2.1	0.24	24.5	36	1.41	FTO/c‐TiO_2_/mp‐TiO_2_/absorber/spiro‐OMeTAD/Au	Spin coating	SnF_2_	59
		FASnI_2_Br	1.72	0.47	6.82	54.3	1.68	ITO/PEDOT:PSS/absorber/C_60_/Ca/Al	Spin coating solvent‐engineering (CB)	No	60
		FASnBr_3_	–	–	–	–	2.4	–	–	No	61
		FA_1−_ *_x_*MA*_x_*SnBr_3_	–	–	–	–	2.4–1.9	–	–	No	
		FA_0.25_MA_0.75_SnI_3_	4.49	0.48	20.7	45.2	1.28	ITO/PEDOT:PSS/absorber/C_60_/BCP/Ag	Spin coating solvent‐engineering (CB)	SnF_2_	28
		FA_0.50_MA_0.50_SnI_3_	5.92	0.53	21.3	52.4	1.3	ITO/PEDOT:PSS/absorber/C_60_/BCP/Ag	Spin coating solvent‐engineering (CB)	SnF_2_	28
		FA_0.75_MA_0.25_SnI_3_	8.12	0.61	21.2	62.	1.33	ITO/PEDOT:PSS/absorber/C_60_/BCP/Ag	Spin coating solvent‐engineering (CB)	SnF_2_	28
		FA_0.8_Cs_0.2_SnI_3_	1.4	0.24	16.1	36	–	ITO/PEDOT:PSS/absorber/PCBM/Bis‐C_60_/Ag	Spin coating solvent‐engineering (Tol)	No	54
		{en}FASnI_3_	7.14	0.48	22.5	66.0	1.5	FTO/c‐TiO_2_/mp‐TiO_2_/absorber/PTTA/Au	Spin coating	SnF_2_	62
		(BA)_2_(MA)_3_Sn_4_I_13_	2.53	0.229	24.1	45.7	1.42	FTO/c‐TiO_2_/mp‐TiO_2_/absorber/PTTA/Au	Spin coating	(TEP)SnF_2_	63
		(PEA)_2_(FA)_8_Sn_9_I_28_	5.94	0.59	14.4	69	1.789	ITO/NiO*_x_*/absorber/PCBM/Al	Spin coating solvent‐engineering (Tol)	SnF_2_	64
		CsSnI_3_	4.81	0.38	25.71	49.1	1.3	FTO/c‐TiO_2_/mp‐TiO_2_/absorber/PTTA/Au (TPFB)	Spin coating	SnI_2_	65
		CsSnI_3_	3.56	0.50	9.89	68	1.3	ITO/absorber/PC_61_BM/BCP/Al	Spin coating	SnCl_2_	66
		CsSnI_3_	3.31	0.52	10.2	62.5	1.3	ITO/NiO*_x_*/absorber/PCBM/Al	Spin coating; coarse‐Grained	‐	67
		CsSnI_3_	2.76	0.43	12.3	39.5	1.3	ITO/CuI/absorber/ICBA/BCP/Al	Spin coating	SnI_2_	68
		CsSnI_3_	2.0	0.24	22.7	37	1.3	FTO/c‐TiO_2_/mp‐TiO_2_/absorber/m‐MTDATA/Au	Spin coating	SnF_2_	69
		CsSnI_3_	1.83	0.17	30.8	34.9	1.25	FTO/c‐TiO_2_/mp‐TiO_2_/absorber/PTTA/Au (TPFB)	Spin coating (hydrazine vapor)	SnF_2_	41
		CsSnI_3_	1.66	0.20	27.7	29	1.27	FTO/TiO_2_/mp‐TiO_2_/absorber/spiro‐OMeTAD/Au	Spin coating	SnF_2_	70
		CsSnI_3_	0.88	0.42	4.8	22	1.3	ITO/absorber/Au/Ti	sequential thermal evaporation	No	71
		CsSnI_3_ a)	8.51	–	–	–	–	HTM in DSSCs	‐	SnF_2_	72
		CsSnI_2_Br	1.67	0.29	15.1	38	1.37	FTO/TiO_2_/mp‐TiO_2_/absorber/spiro‐OMeTAD/Au	Spin coating	SnF_2_	70
		CsSnIBr_2_	3.2	0.31	17.4	57	1.63	FTO/c‐TiO_2_/Al_2_O_3_/absorber/C	Spin coating	HPA‐ SnF_2_	73
		CsSnIBr_2_	1.56	0.31	11.6	43	1.65	FTO/TiO_2_/mp‐TiO_2_/absorber/spiro‐OMeTAD/Au	Spin coating	SnF_2_	70
		CsSnBr_3_	3.04	0.37	14.0	59.4	1.79	FTO/c‐TiO_2_/mp‐TiO_2_/absorber/PTTA/Au (TPFB)	Spin coating (hydrazine vapor)	SnF_2_	41
		CsSnBr_3_	2.17	0.42	9.1	57	1.75	FTO/c‐TiO_2_/mp‐TiO_2_/absorber/spiro‐OMeTAD/Au	Spin coating	SnF_2_	74
		CsSnBr_3_	0.95	0.41	3.99	58	1.75	FTO/TiO_2_/mp‐TiO_2_/absorber/spiro‐OMeTAD/Au	Spin coating	SnF_2_	70
		CsSnBr_3_	0.55	0.45	2.4	55	1.8	ITO/MoO_3_/absorber/C_60_/BCP/Ag	All vapor‐deposited	SnF_2_	75
Sn(IV)		Cs_2_SnI_6_	0.96	0.51	5.41	35	1.48	FTO/TiO_2_/absorber/P3HT/Ag	sequential evaporation	No	76
		Cs_2_SnI_6_	0.86	0.52	3.20	51.5	1.48	FTO/seed layer/ZnO nanorods/absorber/ P3HT/Ag	Spin coating	No	77
		Cs_2_SnI_6_	–	–	–	–	1.3	–	–	–	78
		Cs_2_SnI_6_	–	–	–	–	1.6	–	–	–	79
		Cs_2_SnI_6_	1.47	0.37	6.75	59.5	1.30	FTO/bl‐TiO_2_/2wt% Sn‐TiO_2_/Cs_2_SnI_6−_ *_x_*Br*_x_*/ Cs_2_SnI_6_ HTM/LPAH/FTO	–	No	80
		Cs_2_SnI_5_Br	1.60	0.44	6.58	55.0	1.38				
		Cs_2_SnI_4_Br_2_	2.03	0.56	6.23	57.7	1.40				
		Cs_2_SnI_2_Br_4_	1.08	0.58	3.41	54.8	1.63				
		Cs_2_SnIBr_5_	0.002	0.57	0.01	37.2	2.36				
		Cs_2_SnIBr_6_	non	non	non	non	2.85				
		Cs_2_SnBr_6_	–	–	–	–	2.7	–	–	–	81
		Cs_2_SnCl_6_	–	–	–	–	3.9	–	–	–	
Ge		MAGeI_3_	0.2	0.15	4.0	30	2.0	FTO/c‐TiO_2_/mp‐TiO_2_/absorber/spiro‐OMeTAD/Au	Spin coating	–	–
		FAGeI_3_	–	–	–	–	2.35	‐			
		CsGeI_3_	0.01	0.07	5.7	27	1.63	FTO/c‐TiO_2_/mp‐TiO_2_/absorber/spiro‐OMeTAD/Au			
Bi	0D	MA_3_Bi_2_I_9_	0.42	0.67	1.00	62.5	2.1	ITO/TiO_2_/mp‐TiO_2_/absorber/spiro‐OMeTAD/MoO_3_ /Ag	Spin coating	–	82
		MA_3_Bi_2_I_9_	0.39	0.83	1.39	34	2.22	ITO/PEDOT: PSS/absorber/C_60_/BCP/Ag	Two‐step evaporation–spin‐coating	–	83
		MA_3_Bi_2_I_9_	0.36	0.65	1.10	0.50	2.1	FTO/TiO_2_/mp‐TiO_2_/absorber/spiro‐OMeTAD/Au	Solvent‐engineering (chlorobenzene)		84
		MA_3_Bi_2_I_9_	0.31	0.51	0.94	0.61	–	FTO/TiO_2_/mp‐TiO_2_/absorber/spiro‐OMeTAD/Au	Spin coating	NMP	85
		MA_3_Bi_2_I_9_	0.26	0.56	0.83	49	–	FTO/TiO_2_/mp‐TiO_2_/absorber/spiro‐OMeTAD/Au	Spin coating	–	86
		MA_3_Bi_2_I_9_	0.19	0.35	1.16	46.4	2.11	FTO/TiO_2_/mp‐TiO_2_/absorber/P3HT/Au	Spin coating	–	87
		MA_3_Bi_2_I_9_	0.12	0.68	0.52	33	2.1	FTO/TiO_2_/mp‐TiO_2_/absorber/spiro‐OMeTAD/Ag	Spin coating	–	88
		MA_3_Bi_2_I_9_	0.11	0.72	0.49	31.8	2.26	FTO/TiO_2_/absorber/spiro‐MeOTAD/Au	solvent‐engineering (chlorobenzene)	–	89
		MA_3_Bi_2_I_9_	0.08	0.69	0.37	32	2.1	FTO/TiO_2_/absorber/spiro‐MeOTAD/Ag	Spin coating, gas‐assisted	–	90
		MA_3_Bi_2_I_9_	0.07	0.66	0.22	49	2.9	ITO/PEDOT:PSS/absorber/PCBM/Ca/Al	Spin coating	–	91
		MA_3_Bi_2_I_9_	–	–	–	–	2.04	–	vapor‐assisted conversion	–	92
		MA_3_Bi_2_I_9_Cl*_x_*	0.003	0.04	0.18	38	2.4	FTO/TiO_2_/mp‐TiO_2_/absorber/spiro‐OMeTAD/Ag	Spin coating	–	88
		MA_3_Bi_2_I_9_S*_x_*	–	–	–	–	1.45	–	In situ, thermal	–	93
		(MA_3_Bi_2_I_9_)_0.2_(BiI_3_)_0.8_	0.08	0.57	0.27	50	‐	FTO/TiO_2_/mp‐TiO_2_/absorber/PTAA/PIDT‐DFBT/Ag	Spin coating	–	94
		FA_3_Bi_2_I_9_	–	–	–	–	2.0	–	–	–	95
		(C_3_H_5_N_2_)_3_Bi_2_I_9_	–	–	–	–	–	–	–	–	96
		(C_6_H_14_N)_3_Bi_2_I_9_	–	–	–	–	–	–	–	–	97
	1D	MA_3_Bi_2_Cl_9_	–	–	–	–	–	–	–	–	98
		C_5_H_6_NBiI_4_	0.9	0.62	2.71	0.54	1.98	FTO/c‐TiO_2_/mp‐TiO_2_/absorber/ZrO_2_/C	Spin coating	–	99
		C_6_H_8_NBiI_4_	–	–	–	–	2.17				
		(H_3_NC_6_H_12_NH_3_)BiI_5_	0.03	0.40	0.12	43	2.1	FTO/c‐TiO_2_/mp‐TiO_2_/absorber/spiro‐OMeTAD/Au	Spin coating	–	100
		(TMP)BiX_5_ (X = Cl, Br, I)	–	–	–	–	2.02‐ 3.21	–	–	–	101
	2D	MA_3_Bi_2_Br_9_	–	–	–	–	2.5	–	–	–	102
		(NH_4_)_3_Bi_2_I_9_	–	–	–	–	2.04	–	–	–	103
		(TMP)_1.5_Bi_2_I_7_Cl_2_	–	–	–	–	2.1	–	–	–	101
	3D	MA_2_KBiCl_6_	–	–	–	–	3.04	–	–	–	104
		MA_2_TlBiBr_6_	–	–	–	–	2.16(direct)	–	–	–	105
		MA_2_AgBiBr_6_	–	–	–	–	2.02	–	–	–	106
	0D	Cs_3_Bi_2_I_9_	0.02	0.02	0.18	37	2.03	FTO/TiO_2_/mp‐TiO_2_/absorber/P3HT/Ag	Spin coating	–	100
		Cs_3_Bi_2_I_9_	1.09	0.85	2.15	60	2.2	FTO/TiO_2_/mp‐TiO_2_/absorber/spiro‐OMeTAD/Ag	Spin coating	–	88
		Cs_3_Bi_2_I_9_	–	–	–	–	1.9	–	–		36
		CsBi_3_I_10_	0.40	0.31	3.4	38	1.77	FTO/TiO_2_/mp‐TiO_2_/absorber/P3HT/Ag	Spin coating	–	100
	2D	K_3_Bi_2_I_9_	–	–	–	–	2.1	–	–	–	36
		Rb_3_Bi_2_I_9_	–	–	–	–		–	–	–	
		Cs_3_Bi_2_Br_9_	–	–	–	–	2.71	–	–	–	107
	3D	Cs_2_AgBiBr_6_	–	–	–	–	1.9	–	–	–	108
		Cs_2_AgBiBr_6_	–	–	–	–	1.95		–	–	38
		Cs_2_AgBiBr_6_	–	–	–	–	2.19	–	–	–	109
		Cs_2_AgBiBr_6_	2.43	0.98	3.93	63	2.21	FTO/c‐TiO_2_/mp‐TiO_2_/absorber/spiro‐OMeTAD/Au	Spin coating	–	110
		Cs_2_(Ag_1−_ *_a_*Bi_1−_ *_b_*)Tl*_x_*Br_6_	–	–	–	–	1.40 1.57(direct)	–	–	–	111
		Cs_2_Ag(Bi_0.625_Sb_0.375_)Br_6_	–	–	–	–	1.86 2.15(direct)	–	–	–	112
		Cs_2_AgBiCl_6_	–	–	–	–	2.2	–	–	–	108
		Cs_2_AgBiCl_6_	–	–	–	–	2.77	–	–	–	109
		Cs_2_InAgCl_6_ (non‐Bi)	–	–	–	–	3.3(direct)	–	–	–	113
		AgBi_2_I_7_	1.22	0.56	3.30	67.4	1.87	FTO/TiO_2_/mp‐TiO_2_/absorber/P3HT/Ag	Spin coating	–	114
		Ag_2_BiI_5_ (R$\bar{3}$ m AgI/BiI_3_ = 2: 1)	2.1	0.49	6.8	63	1.85	FTO/TiO_2_/mp‐TiO_2_/absorber/P3HT/Au	Spin coating	–	115
		AgBi_2_I_7_ (Fd$\bar{3}$ m AgI/BiI_3_ = 1: 2)	0.4	0.46	1.6	56	1.78				
Sb		(NH_4_)_3_Sb_2_I*_x_*Br_9−_ *_x_*	0.51	1.03	1.15	42.9	2.27‐ 2.78	ITO/PEDOT:PSS/absorber/PC_61_BM/Al	Spin coating	–	116
		MA_3_Sb_2_I_9_	0.49	0.90	1.0	55	2.14	ITO/PEDOT:PSS/absorber/PC_61_BM/nano‐ZnO/Al	Spin coating; solvent‐engineering (Tol)	–	117
		MA_3_Sb_2_I_9_	2.04	0.62	5.41	60.8	1.95	ITO/PEDOT:PSS/absorber/PC_61_BM/ C_60_/BCP/Al	Spin coating	HI	118
		Rb_3_Sb_2_I_9_	0.66	0.55	2.11	57	2.1	FTO/TiO_2_/mp‐TiO_2_/absorber/Poly‐TPD/ Au	SbI_3_ in toluene treated (Tol)	SbI_3_	35
		Cs_3_Sb_2_I_9_	<1.0	0.31	<0.1	–	2.05	FTO/c‐TiO_2_/ absorber/PTAA/Au	Co‐evaporation / vapor‐assisted conversion	SbI_3_ vapor	119
		Cs_3_Sb_2_I_9_	0.84	0.6	2.91	48.1	2.0	ITO/PEDOT:PSS/absorber/PC_61_BM/ C_60_/BCP/Al	Spin coating	HI	118
		[CH_3_SC(NH_2_)_2_]_2_SbA_5_	–	–	–	–	2.41–3.34	–	–	–	120
		Cs_4_CuSb_2_Cl_12_	–	–	–	–	1.0	–	–	–	121
Cu		(CH_3_(CH_2_)_3_NH_3_)_2_CuBr_4_	0.63	0.88	1.78	40	1.76	FTO/c‐TiO_2_/mp‐TiO_2_/absorber/spiro‐OMeTAD/Ag	Spin coating	–	122
		(*p*‐F‐C_6_H_5_C_2_H_4_‐NH_3_)_2_‐CuBr_4_	0.51	0.87	1.46	40	1.74				
		MA_2_CuCl*_2_*Br_2_	0.02	0.26	0.22	32	2.12	FTO/c‐TiO_2_/mp‐TiO_2_/absorber/spiro‐OMeTAD/Au	Spin coating	–	39
		MA_2_CuCl*_0.5_*Br_3.5_	0.002	0.29	0.021	28	1.8				
		C_6_H_4_NH_2_CuBr_2_I	0.46	0.20	6.20	46	1.64	FTO/c‐TiO_2_/mp‐TiO_2_/absorber/ZrO_2_/C	Drop casting	–	123

^a)^ Used as an HTM in the solid‐state dye‐sensitized solar cell.

## 2 Lead‐Free Halide Hybrid Perovskite and Related Absorbers

Although we have achieved high efficiency beyond 22% based on lead halide perovskites, which is comparable to commercial crystalline silicon solar cells, the existence of Pb is an urgent problem against the final application of PSCs. The toxicity of lead is documented to disturb the functioning of the blood, kidneys, liver, testes, brain, and nervous system.^124^, ^125^, ^126^ The toxicity of lead is due, in general, to its binding affinity to thiol and cellular phosphate groups of numerous enzymes, proteins, and cell membranes.^127^ Lead is toxic to the central nervous system, especially in children.^128^ Thus, there come the questions that: can we eat the cake and have it too? Meaning can we have high‐efficiency PSCs without being poisoned? The urge to explore authentic high‐efficiency lead‐free metal halide absorbers led to an abundance of works which will be shown in the following sections.

### 2.1 The Group 14 Metals (Sn and Ge) Based Absorbers

#### 2.1.1 Tin‐Based Absorbers

Tin (Sn) as a member of group 14 congeners and less‐toxic metal (than Pb) is expected to have comparable properties with its Pb analogs. Owing to their direct and narrow bandgaps (1.2–1.4 eV), low exciton binding energies (18 meV), and super charge‐carrier mobilities,^129^, ^130^, ^131^, ^132^ Sn‐based perovskites ASnX_3_ (A = MA, FA or Cs, X = halide) were the most investigated nonlead hybrid perovskite absorbers. Depending on the types of A cations and the solid structure, we classified them into following groups and made discussions accordingly.

##### MASnI_3_

**Figure 3:**
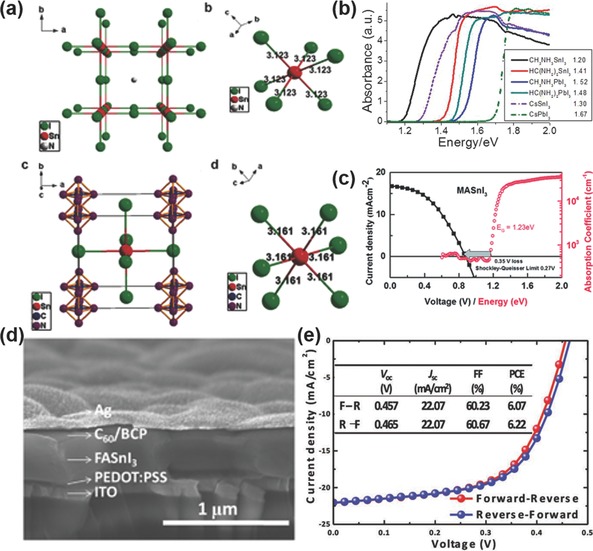
a) Ball‐and‐stick diagrams of crystal structures and the {SnI_6_} octahedral structure units in the MASnI_3_ and FASnI_3_ single crystals. Reproduced with permission.^133^ b) The spectra of MASnI_3_ and FASnI_3_ prepared with the solution method as compared with other perovskites. Reproduced with permission.^132^ Copyright 2013, ACS. c) *J*–*V* curve of MASnI_3_ on TiO_2_ and bandgap of the material determined using the Tauc plot. Reproduced with permission.^46^ Copyright 2014, RSC. d) Cross‐sectional SEM image of the entire device with FASnI_3_ and 10 mol% SnF_2_ additive; e) *J*–*V* characteristics of FASnI_3_‐based devices under 100 mW cm^−2^ AM1.5G illumination under reverse and forward voltage scans. Reproduced with permission.^56^

The crystal structure of MASnI_3_ belongs to the cubic $Pm\bar{3}$ *m* (no. 221) space group at room temperature (**Figure** **3**a). MASnI_3_ possesses an optical bandgap of 1.20–1.35 eV (Figure 3b), displaying remarkably high carrier mobility (electron mobility ≈2320 cm^2^ V^−1^ s^−1^, hole carrier mobility of ≈322 cm^2^ V^−1^ s^−1^),^132^, ^133^ and long carrier diffusion lengths exceeding 500 nm.^134^ In 2014, MASnI_3_ 46 and MASn(I_1−_ *_x_*Br*_x_*)_3_ 51 were reported as the first completely lead‐free solar absorbers in PSCs based on a traditional mesoscopic device structure of FTO/c‐TiO_2_/mp‐TiO_2_/absorber/spiro‐OMeTAD/Au. PCEs of 6.4% and 5.73% were reported, respectively, and after three years they are still the record efficiencies among MASnX_3_‐based PSCs (Figure 3c). This dramatic different situation from the lead‐based perovskite can be understood by the fact that Sn‐based perovskites are prone to self‐doping in ambient air resulting in instability and poor reproducibility.^46^, ^47^, ^48^, ^51^, ^52^, ^53^, ^135^

##### FASnI_3_

**Figure 4:**
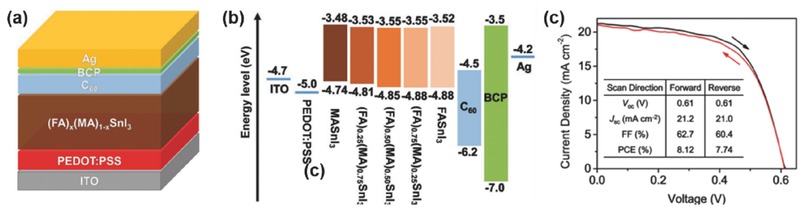
a) Schematic illustration of the device structure. b) Band alignment diagram. c) *J*–*V* curves of the champion device measured using both forward and reverse scan mode at a scan rate of 300 mV s^−1^ under the simulation of AM 1.5G, 100 mW cm^−2^. Reproduced with permission.^28^

Similar to the case of lead halide perovskite, FA is another candidate that can be employed as A site cation for Sn halide perovskites. FASnI_3_ takes the cubic $Pm\bar{3}$ *m* (no. 221) space group structure at room temperature and has only one single stable phase up to 200 °C (Figure 3a). This is different from its lead analogue‐FAPbI_3_, which has two competing δ‐phase and α‐phase structure.^132^, ^136^, ^137^ Another unique point is that due to the symmetry reduction of the 3D [SnI_3_]^−^ framework with larger cations group like FA,^132^ FASnI_3_ has a larger band gap (1.41 eV) value than that of MASnI_3_.^59^ In 2015, Koh et al. first took FASnI_3_ as a light absorber in solar cell applications and realized a high short‐circuit current density (*J*_SC_) of 24.45 mA cm^−2^ and PCE of merely 2.1%.^59^ It was reported that FASnI_3_ is more stable than MASnI_3_ due to the reduced extent of Sn oxidation_._ 133, 138 Accordingly, FASnI_3_‐based PSCs exhibited much better reproducibility as compared to MASnI_3_‐based devices.^138^ In 2016, Liao et al.^56^ reported a PCE of 6.22% based on FASnI_3_ PSCs with high reproducibility, which is one of the best efficiencies among Sn‐based PSCs (Figure 3d,e). Very recently, Shi et al.^139^ studied the phenomenon theoretically and found that the antibonding coupling between Sn‐s and I‐p is weaker in FASnI_3_ than in MASnI_3_ due to the larger ionic size of FA, leading to higher formation energies of Sn vacancies in FASnI_3_. Subsequently, the conductivity of FASnI_3_ can be tuned from p‐type to intrinsic by varying the growth conditions of the perovskite semiconductor. In contrast, MASnI_3_ shows unipolar high p‐type conductivity independent of the growth conditions. Ion mixing is also one important approach for Sn perovskites. Ferrara et al.^61^ reported the first mixed A‐cation compositions of tin perovskites FA_1‐_ *_x_*MA*_x_*SnBr_3_, with cubic structures and the band gaps ranging from 2.4 eV (*x* = 0) to ≈1.92 eV (*x* = 0.82). However, no device performance was reported. Very recently, Zhao et al.^28^ reported another mixed‐organic‐cation perovskite absorber (FA)*_x_*(MA)_1−_ *_x_*SnI_3_. The optimized ratio of FA and MA cations is 0.75 versus 0.25 and the resulted (FA)_0.75_(MA)_0.25_SnI_3_ has an ideal bandgap of 1.33 eV. PSCs with an inverted structure based on (FA)_0.75_(MA)_0.25_SnI_3_ showed an improved *V*_OC_ of 0.61 V and the best ever PCE of 8.12%, which is the highest efficiency among the Sn‐based PSCs (**Figure** **4**).

##### CsSnI_3_

**Figure 5:**
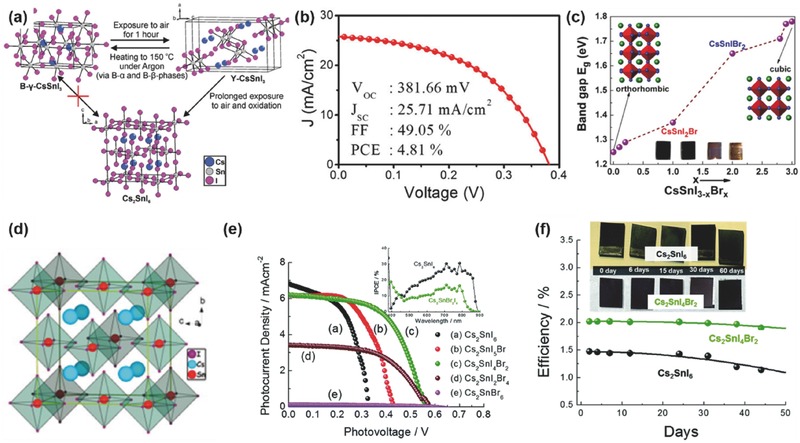
a) Crystal structures of B‐Y‐CsSnI_3_, Y‐CsSnI_3,_ and Cs_2_SnI_6_ and their phase transformation routes. Reproduced with permission.^143^ Copyright 2017, ACS. b) *J*–*V* characteristic of the 0.4‐CsI/SnI_2_‐based device. Reproduced with permission.^65^ Copyright 2017, ACS. c) Bandgap variation with Br concentration. Reproduced with permission.^70^ Copyright 2015, ACS. d) Distorted 3D structure of Cs_2_SnI_6_ at room temperature. Reproduced with permission.^78^ Copyright 2014, ACS. e) *J–V* curves of a series of cells with different composition of Cs_2_SnI_6_ *_−x_*Br*_x_*. The inset shows the IPCE values. f) The stability curves for 50 d are shown here for Cs_2_SnI_6_ (black), and the Cs_2_SnBr_2_I_4_ (green)‐based solar cells. Reproduced with permission.^80^ Copyright 2017, RSC.

Compared to the organic–inorganic hybrid perovskite materials, the all‐inorganic halide perovskites exhibited much higher thermal stability.^16^, ^70^, ^140^ Replacing the organic cations (MA^+^ or FA^+^) with the inorganic Cs^+^, CsSnI_3_ perovskite shows a melting point of 435 °C,^72^, ^141^ suggesting superior intrinsic thermal stability. In contrast, hybrid perovskites MASnI_3_ and FASnI_3_ start to decompose at ≈200 °C. CsSnI_3_ adopts a black orthorhombic perovskite phase structure, possesses a direct band gap of 1.3 eV, and has a high hole mobility of ≈585 cm^2^ V^−1^ s^−1^ at room temperature (**Figure** **5**a). In 2012, due to its high hole mobility, it was first taken as a hole transport material (HTM) in DSSCs.^72^ Very recently, the further potential of CsSnI_3_ for solar cell applications was uncovered by Wu et al.^142^ They reported that the melt‐synthesized CsSnI_3_ ingots contain high‐quality large single crystal (SC) grains, which bear superior properties like high bulk carrier lifetimes (>6.6 ns), low doping concentrations of ≈4.5 × 10^17^ cm^−3^, and long minority‐carrier diffusion lengths approaching 1 µm. In this regard, they predicted a PCE of ≈23% for optimized CsSnI_3_ SC solar cells. As a light absorber, CsSnI_3_ was firstly used in a Schottky contact type PSC with a simple layer structure of ITO/CsSnI_3_/Au/Ti in 2012, showing a PCE of 0.9%.^71^ Four years later, Marshall et al.^66^ designed an HTM‐free CsSnI_3_ PSC with exceptionally high fill factor up to 0.69, and a PCE up to 3.56%. In 2017, Song et al.^65^ assembled CsSnI_3_‐based PSCs with reducing atmosphere‐assisted dispersible additive. The new technique produced a PCE of up to 4.81%, which is the highest efficiency among all CsSnI_3_ PSCs so far (Figure 5a). Meanwhile, benefiting from the superior thermal stability of CsSnX_3_ perovskite, Li et al.^73^ fabricated an all‐inorganic CsSnIBr_2_ mesoscopic PSC with thermal stability up to 200 °C, achieving an average PCE of 3.0% and long‐term stability without efficiency‐loss over 77 d (Figure 5c).

##### Cs_2_SnI_6_

The Sn^II^ in CsSnI_3_ and M(F)ASnI_3_ has the tendency to be oxidized to Sn^IV^ leading to the spontaneous change to Cs_2_SnI_6_ and M(F)A_2_SnI_6_ respectively in ambient atmosphere. Cs_2_SnI_6_ with a molecular salt structure is not bona fide perovskite but crystallizes into a face‐centered cubic $Pm\bar{3}$ *m* space group and the lattice parameter of which is 11.63 Å^132^, ^144^ (Figure 5d). The direct bandgap (*E*_g_) of Cs_2_SnI_6_ is ≈1.3–1.6 eV^76^, ^79^, ^132^ depending on the preparation methods at room temperature with absorption coefficient over 10^5^ cm^−1^ from 1.7 eV. Computational work based on Cs_2_SnI_6_ 145 shows that the iodine vacancies and interstitial Sn are the dominant defects that give intrinsic n‐type behavior, unlike the p‐type behavior in CsSnI_3_. The carrier concentration (electrons) of Cs_2_SnI_6_ is on the order of ≈1 × 10^14^ cm^−3^ measured by Hall effect measurements at room temperature, with a high electron mobility of 310 cm^2^ V^−1^ s^−1^. Moreover, due to the full oxidation state of Sn^4+^, Cs_2_SnI_6_ exhibits better stability in the air with moisture than CsSnI_3_. In 2014, Lee et al. used Cs_2_SnI_6_ as a hole transporter in solid‐state DSSCs and fabricated devices in the air with an efficiency close to 8%.^78^ The study of Cs_2_SnI_6_ as a light absorber in the solar cells is rare. Until 2016, Cs_2_SnI_6_ was first studied by Qiu et al.^76^, ^77^ as a light harvester, demonstrating a PCE of ≈1%. Very recently, Lee et al.^80^ further improved the PCE up to 1.47%. They designed a series of compounds with the general formula of Cs_2_SnI_6−_ *_x_*Br*_x_*. With the increase of Br composition (*x*), the bandgaps can be tuned from ≈1.3 to ≈2.9 eV and the color of the films was changing from dark brown to brown/red, then to light yellow. The Cs_2_SnI_6−_ *_x_*Br*_x_* films were fabricated with a two‐step solution process: the crystal structure of CsI was optimized in Step‐1 by postannealing at 300 °C for 30 min after electrospraying deposition and in Step‐2 the CsI film was reacted with a SnI_4_ solution at 110 °C for 20 min (Figure 5e,f). After that, a stoichiometric, smooth, uniform, and thick active layer was obtained. The freshly made films were constructed into the typical “sandwich” type device structure of FTO/bl‐TiO_2_/2wt% Sn‐TiO_2_/Cs_2_SnI_6‐_ *_x_*Br*_x_*/Cs_2_SnI_6_ HTM/large‐effective‐surface‐area polyaromatic hydrocarbon (LPAH)/FTO. The best‐achieved efficiency was ≈2.03% when *x* = 2. It is worth noting that their device fabrication process was carried out in the air and did not use any additive to protect the active material. The Cs_2_SnI_6−_ *_x_*Br*_x_* films and corresponding devices showed excellent stability in the air for 50 d (see Figure 5f). Thus, being a molecular salt (0D), Cs_2_SnI_6_ has the bandgap similar to that of the 3D perovskites (CsSnI_3_ and MAPbI_3_), high absorption coefficient, and high carrier mobility. Coupled with its intrinsic ambient stability, such Sn‐perovskite variants can be explored with more effort in the future for achieving more efficient and stable Sn‐based PSCs.

##### (PEA)_2_(FA)_8_Sn_9_I_28_

In the attempt to improve the environmental stability, Cao et al.^63^ decrease the dimensionality of the perovskite materials by mixing the CH_3_(CH_2_)_3_NH_3_^+^ (BA^+^) and CH_3_NH_3_^+^ (MA^+^). The obtained 2D Ruddlesden−Popper (CH_3_(CH_2_)_3_NH_3_)_2_(CH_3_NH_3_)*_n_*_−1_Sn*_n_*I_3_ *_n_*_+1_ perovskites possessed optimal optical bandgaps of 1.50 and 1.42 eV for solar cells when *n* = 3 and *n* = 4, respectively. The 2D tin perovskite outperformed its 3D analogs for higher moisture stability with an encouraging PCE of 2.5% (from *n* = 4). More importantly, incorporating 20% phenyl ethyl ammonium (PEA) into FA‐based Sn iodide perovskites yielded low dimensional (PEA)_2_(FA)_8_Sn_9_I_28_ 64 which exhibited markedly enhanced air stability in comparison with their 3D counterparts FASnI_3_. The inverted structure‐based devices with (PEA)_2_(FA)_8_Sn_9_I_28_ perovskite exhibited the best PCE up to 5.94% and showed super stability over 100 h without encapsulation.

#### 2.1.2. Germanium‐Based Absorbers

**Figure 6:**
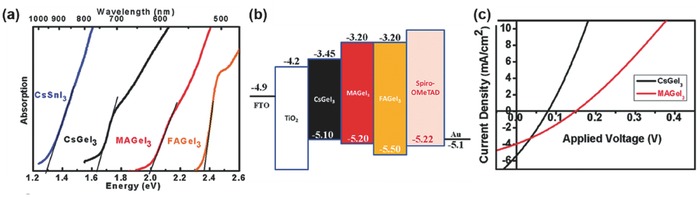
a) Optical absorption spectrum of CsGeI_3_, MAGeI_3,_ and FAGeI_3_, in comparison with CsSnI_3_; b) Schematic energy level diagram of CsGeI_3_, MAGeI_3,_ and FAGeI_3_; c) *J*–*V* curves of photovoltaic devices fabricated with different germanium halide perovskites. Reproduced with permission.^40^ Copyright 2015, RSC.

Germanium, another group IV metal, with ns^2^ electronic configuration, has the same valent state with the lead. Due to the 4s lone pairs of Ge is more active than the Pb 6s lone pair, Ge^2+^ is easier to be oxidized leading to metallic conductivity in the hybrid materials and short‐circuit behavior in the photovoltaic devices similar to the case of Sn‐based perovskite.^40^, ^135^, ^141^ However, germanium has demonstrated much less toxicity compared to lead^146^ and is expected to be a promising candidate in the search for Pb‐free perovskite materials. To prove the concept, Sun et al.^147^ investigated the structural and electronic properties of MAGeX_3_ (X = Cl, Br, I) by density functional theory (DFT) methods and showed that MAGeI_3_ is a good absorber for applications in PSCs. Based on DFT calculation, Ming et al.^148^ also proposed that CsGeI_3_ might be a good HTM in solar cells. Krishnamoorthy^40^ studied the solid structure of three AGeI_3_ (A = Cs, MA, or FA) halide perovskites and revealed trigonal phase (with *R*3*m* space group symmetry) in contrast to Pb and Sn‐based perovskites with a tetragonal phase (*I*4/*mcm*)^147^ at room temperature. All compounds are remarkably stable up to 150 °C and show no phase transition in the range of device working temperatures (r.t. to 150 °C). With increasing size of the A^+^ cation, the band gaps of AGeI_3_ are 1.63, 2.0, and 2.35 eV for CsGeI_3_, MAGeI_3_, and FAGeI_3_, respectively (**Figure** **6**a). The values of the valence bands of CsGeI_3_, MAGeI_3,_ and FAGeI_3_ perovskites measured by the PESA are −5.10, −5.2, and −5.5 eV, respectively (Figure 6b). However, unfortunately, the solar cells based on AGeI_3_ were fabricated with a mesoscopic structure, achieving 0.2% PCE for MAGeI_3_, 0.11% PCE for CsGeI_3_ and no photocurrent for FAGeI_3_ (Figure 6c). The low performance of AGeI_3_‐based solar cells was attributed to the low concentration of precursor solutions, poor quality of perovskite films, and the oxidation sensitivity of the materials. Recently, theorists studied mixed tin and germanium perovskites and predicted that RbSn_0.5_Ge_0.5_I_3_ possesses not only a direct bandgap within the optimal range of 0.9–1.6 eV but also a desirable optical absorption spectrum that is comparable to those of the state‐of‐the‐art MAPbI_3_ perovskites. It has favorable effective masses for high carrier mobility as well as a greater resistance to water penetration than the prototypical inorganic–organic lead‐containing halide perovskite.^149^

#### 2.1.3. Devices Engineering Efforts Toward High Efficiency and Stable Sn^2+^‐Based PSCs

Thanks to the great endeavor of the researchers, Sn‐based PSCs have obtained relative stable PCE of ≈8%.^28^ The improvement in performance of PSCs is not only due to the material itself but also the evolution in device engineering. In this small section, we will introduce various devices preparation approaches towards efficient and stable Sn^2+^‐based PSCs over the recent years.

##### 2.1.3.1. Efforts to Suppress Sn Vacancy Defects and Improve Oxidation Stability:

One of the major problems of Sn‐based PSCs is their poor stability originating from the facile formation of Sn vacancy associated with oxidation of Sn^2+^ to Sn^4+^ when exposed in air,^46^, ^141^, ^150^ which leads to poor reproducibility and deteriorates the devices rapidly.^46^, ^51^ For example, Xu et al.^151^ studied the defect properties of CsSnI_3_ perovskite and depicted the influence of defects and synthesis conditions on the photovoltaic performance. They found that due to the strong Sn 4s‐I 5p antibonding coupling, Sn vacancies have very low formation energies in CsSnI_3_, leading to a very high concentration of Sn vacancies and therefore high p‐type conductivity regardless of the growth conditions. To solve the problem, reductive or sacrificial additives like Sn(II) halide salt etc., were added to cure the problem.

##### SnF_2_

**Figure 7:**
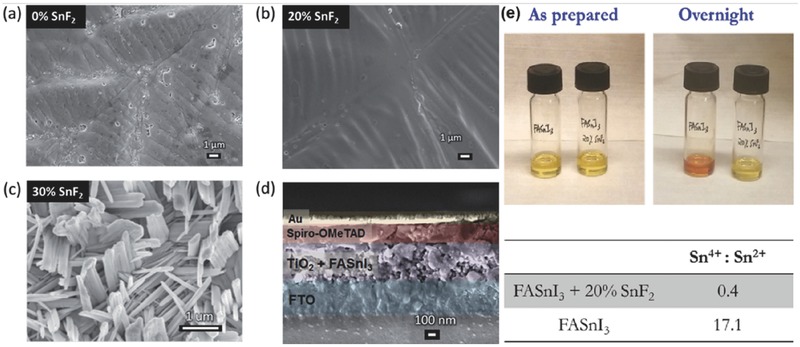
a,b) Top‐view FESEM images of FASnI_3_ and FASnI_3_:20%SnF_2_ perovskite films deposited on the mesoporous TiO_2_ layer. c) Expanded view of the nanoplatelet‐like structure found on the surface of FASnI_3_:30%SnF_2_. d) Cross‐sectional FESEM image of the full device showing individual layers as follows: FTO/TiO_2_ + FASnI_3_/spiro‐OMeTAD/Au. e) Color change in FASnI_3_ with and without SnF_2_ addition and XPS measurement. Reproduced with permission.^59^ Copyright 2015, RSC.

Before tin halide perovskite served as a light‐absorber in PSCs, Chung et al.^72^ demonstrated that when CsSnI_3_ with SnF_2_ additive (though the formulation of CsSnI_2.95_F_0.05_ is not correct^152^) was used as HTM in DSSC, enhancements of 29% and 21% in *J*_SC_ and η were achieved, respectively. The magic effect of SnF_2_ attracted researcher to investigate its working mechanism. In 2014, Kumar et al.^69^ demonstrated for the first time that the introduction of SnF_2_ into CsSnI_3_ can reduce Sn vacancies and background carrier densities. Therefore, high *J*_SC_ of more than 22 mA cm^−2^ and a PCE of 2.02% were achieved in contrast to the nonfunctioning devices without SnF_2_. One year later, the same group^59^ further investigated the doping effects of SnF_2_ in FASnI_3_. The X‐ray photoelectron spectroscopy (XPS) data show that only Sn^2+^ but no Sn^4+^ appears in the FASnI_3_: 20% SnF_2_ sample, indicating that no oxidation side reaction happened inside the doped sample (**Figure** **7**a–d). Moreover, they claimed that the addition of SnF_2_ improved the environmental stability, as they found that the color of the FASnI_3_ films with SnF_2_ remained yellow when left overnight, but change into orange without SnF_2_ (Figure 7e). The resulting photovoltaic devices gave maximum PCE of 2.10%, with high *J*_SC_ of 24.45 mA cm^−2^. Based on the same hypothesis, SnF_2_ dopant was also introduced to reduce Sn vacancies for efficient and stable CsSnBr_3_ based PSCs.^74^, ^75^ The author has concluded that the SnF_2_ cannot only improve interfacial energy alignment but also increase stability to electron beam damage. Nowadays, most Sn‐based perovskites films prepared by solution deposition need additional SnF_2_ to get good performance,^47^, ^48^, ^57^, ^58^, ^73^ including the recent (FA)_0.75_(MA)_0.25_SnI_3_ based PSCs with the highest PCE of 8.12%.^56^ Additionally, Ma et al.^134^ claimed that SnF_2_ as the inhibitor of Sn^2+^ oxidation in the MASnI_3_ film could increase the fluorescence lifetime up to 10 times and give longer carrier diffusion lengths >500 nm, compared with pristine MASnI_3_ films.

##### SnX_2_ (X = Cl, Br, I)

Apart from the tin fluoride additives, excess tin halide salts like SnI_2_ were also believed to improve device stability towards air oxidation. In 2015, Marshall et al.^68^ prepared the CsSnI_3_ films with an excess of SnI_2_ during CsSnI_3_ synthesis from CsI and SnI_2_. They believed that the excess of SnI_2_ occupies some of the space between adjacent CsSnI_3_ crystals, hindering the ingress of the oxygen and water so that the barrier of the transformation of CsSnI_3_ into Cs_2_CsI_6_ at the site of a Sn vacancy is increased. The fabricated devices have inverted planar structure of ITO/CuI/CsSnI_3_ (with or without the excess of SnI_2_)/C_60_/BCP/Al. The devices with the excess amount of SnI_2_ exhibit up to 30% increase in *J*_SC_ and *V*_OC_ as compared to the control devices. The devices' stability is also enhanced in that the PCE of devices with 10 mol% excess SnI_2_ exhibited only 10% reduction after 10 d period of storage, contrary to the 70% loss with control devices. In 2016, they further demonstrated that excess SnI_2_ is beneficial to improve both efficiency and stability of CsSnI_3_‐based PSCs.^66^ Therefore, their results suggest that the excess amount of SnI_2_ in the precursor is also an effective strategy to improve the performance of CsSnI_3_‐based PSCs. Similarly, an excess of SnBr_2_ 75 was also found to reduce the density of defect states or Sn vacancies in all‐thermal‐vapor deposited CsSnBr_3_ PSCs by preventing the oxidation of Sn^2+^ to Sn^4+^ in ambient air. The resulted devices obtained a higher *V*_OC_ of 0.41 V than the previously reported 0.19 V.^23^

**Figure 8:**
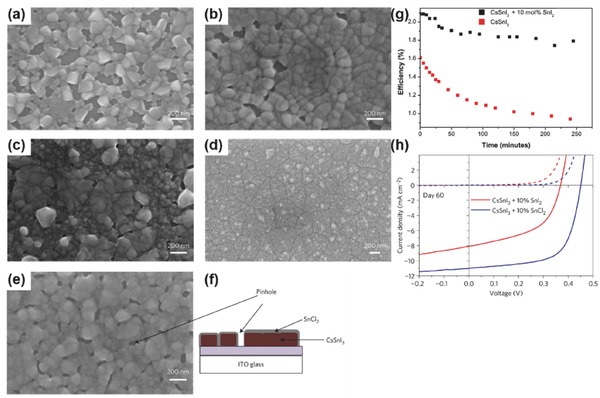
SEM images of CsSnI_3_ films on ITO glass prepared with different tin halide additives. SEM images with no a) tin halide additive, b) 10 mol% added SnI_2_, c) 10 mol% added SnBr_2_, d) 10 mol% added SnF_2_, and e) 10 mol% added SnCl_2_. f) Schematic diagram of the proposed film structure in case e: CsSnI_3_ crystallites capped with a thin SnCl_2_ layer. Reproduced with permission.^66^ Copyright 2016, Nature Publishing Group. g) The effect of constant illumination on PCE of devices (100 mW cm^−2^ of AM 1.5) for 245 min. Reproduced with permission.^68^ Copyright 2015, RSC. h) *J–V* characteristics of different CsSnI_3_ devices after 60 d storage under nitrogen. Reproduced with permission.^66^ Copyright 2016, Nature Publishing Group.

It was not until 2016 that a systematic study was made about the effect of different Tin halide salts in resolving the problem of tin vacancies in tin halide perovskites^66^ (**Figure** **8**a–f). Focusing on the low fill factor (FF) problem of Sn‐based PSCs, Marshall et al. evaluated SnF_2_, SnCl_2_ and SnBr_2_ additives in CsSnI_3_‐based PSCs. Simplified device architecture (ITO/CsSnI_3_/PC_61_BM/BCP/Al) was fabricated without HTM and the performance of SnCl_2_ doped CsSnI_3_ PSC devices was tested without encapsulation in ambient air at a humidity of ≈25% under constant 1 sun simulated solar illumination. They showed that the champion stability was exhibited only by devices with a tin halide additive: 11 h with no additive; 16 h with 10 mol% SnCl_2_; and 22 h for SnI_2_ (Figure 8g,h). Moreover, SnCl_2_ as additive offers the advantage of the highest η (3.56%, due to reduced sensitivity of device parameters to pin holes while SnBr_2_ and SnF_2_ gave η ≤ 0.4%. For the same reason, SnCl_2_‐doped CsSnI_3_ also showed FF up to 0.69, which is the highest FF among reported tin halide PSCs. They attributed SnCl_2_ being the best of the tin halide additives to the interplay of three factors. First, the added SnCl_2_ is distributed toward the surface of the crystallites to form a tin‐rich top layer for the perovskites. Second, the greater covalence offers SnCl_2_ better solubility in common solvents (including DMF) than SnF_2_. Last, tin chloride diffusion into fullerene (ETL) is easier than other halide analogs. Therefore, SnCl_2_ can be used as a promising additive to improve the performance of CsSnI_3_ PSCs and probably any other Sn‐based PSCs. Currently, only one report on SnCl_2_ modified FASnI_3_ as an inhibitor of Sn^4+^, but poor PCE was achieved.^153^

##### SnO and Sn(OH)_2_

Except for the tin halide additives, the presence of SnO and Sn(OH)_2_ 53 in the MASnI_3_ film was also found to be beneficial to reduce hole carrier concentration, leading to an improved air stability of the Sn‐based perovskite devices. This finding suggests that not only the commonly used tin halide additives but also other divalent Sn compounds could serve as Sn vacancy suppressors assisting the realization of efficient and stable tin‐based PSCs.

##### SnX_2_/Organic Reduce Agents

**Figure 9:**
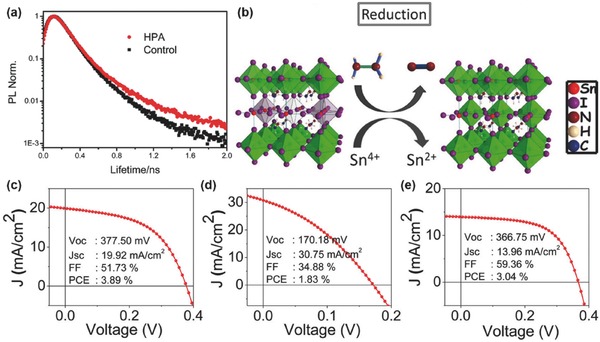
a) PL time decay trace on CsSnIBr_2_ perovskite thin films on glass attached Al_2_O_3_ mesoporous layer. Reproduced with permission.^73^ Copyright 2016, RSC. b) Proposed possible mechanism of hydrazine vapor reaction with Sn‐based perovskite materials. Reduction process: 2SnI_6_^2−^ + N_2_H_4_ → 2SnI_4_^2−^ + N_2_ + 4HI. The best *J–V* curves of c) MASnI_3_, d) CsSnI_3_, and e) CsSnBr_3_. Reproduced with permission.^41^ Copyright 2017, ACS.

The primary cause of the instability of tin‐based PSCs is the oxidation of the Sn(II) to Sn(IV). Therefore, it is reasonable to add reductive agents besides divalent tin additives, to further suppress Sn vacancy for more stable lead‐free PSCs. As an example, hypophosphorous acid (HPA)^73^ (**Figure** **9**a) was introduced into fabricating all‐inorganic CsSnIBr_2_‐based mesoscopic PSCs to reduce the concentration of Sn vacancies, achieving a higher PCE of 3.0% than 1.7% of without HPA addition. Moreover, Song et al.^41^ introduced hydrazine vapor (reducing vapor atmosphere) in the presence of SnF_2_ additive into fabrication process of Sn‐based PSCs (Figure 9b). The results showed that the additional hydrazine vapor process led to significantly suppressed carrier recombination with more than 20% reduction of Sn^4+^/Sn^2+^ ratios. And by tuning amounts of hydrazine vapor properly, the PCEs of MASnI_3_ and CsSnI_3_ devices were improved from an average of ≈0.02% to 3.40% and ≈0.16% to 1.50%, respectively. Four months later, the same group reported^65^ more efficient CsSnI_3_ cells with PCE up to 4.81% using the same hydrazine vapor treatment with an excess of SnI_2_. In this research, they also studied the optimum ratios of monovalence cations (MA^+^, FA^+^_,_ or Cs^+^) to SnI_2_ in the MASnI_3_, FASnI_3_ and CsSnI_3_ perovskite materials, which were 0.4–0.6, 0.6–0.8, and 0.4, respectively. Furthermore, they prepared ASnI_3_ devices in a pure N_2_ atmosphere for comparison. The results showed that only FASnI_3_ could work with *V*_OC_ > 0.15 V, while the other two devices behaved short‐circuit even at the optimum AI/SnI_2_ (A: MA^+^, FA^+^_,_ or Cs^+^) ratio that worked in a weak hydrazine vapor atmosphere. This result showed the importance of using reducing agents like hydrazine to suppress SnI_2_ from forming Sn^4+^ and ensure Sn^2+^‐rich environment to compensate the Sn^2+^ vacancies for obtaining efficient devices. On the other hand, the phenomenon that only FASnI_3_ ‐based devices could survive without additional hydrazine consists with aforementioned results^133^, ^138^ where FASnI_3_ displayed alleviated self‐doping effect possibly due to the competitive formation the hydrogen bonding between H_2_O and FA^+^ (Figure 9c–e).

##### 5‐Ammonium Valeric Acid Iodide and Ascorbic Acid

In the end, beyond above‐mentioned Sn(II) containing panaceas, Hoshi et al.^154^ claimed that the addition of 5‐AVAI in the preparation process could significantly improve the oxidation stability of the MASnI_3_ films in the air, due possibly to the formation of (5‐AVAI)*_x_*(CH_3_NH_3_)_1−_ *_x_*PbI_3_. In addition, ascorbic acid (AA),^155^ as a common antioxidant, was also introduced as an effective additive to retard the oxidation of Sn‐containing precursor solutions for making Pb/Sn mixed perovskites. It is of great necessity to examine these effects on the pure Sn‐based perovskites.

##### 2.1.3.2. Methods to Control the Morphology of the Sn‐Based Perovskite Layers:

Apart from the divalent tin additives, the film quality of the fabricated perovskite layer is another parameter that controls the final performance of the tin‐based PSCs. The film quality mainly refers to the morphology of the perovskite layer, such as homogeneity and coverage, which are strongly influenced by the crystallization process of perovskite film. The conventional one‐step film deposition method of tin perovskites often engenders randomly oriented film growth accompanied by forming large crystals platelets and poor surface coverage with micron‐sized pinholes.^46^ The flawed films further led to the poorly performing devices.^69^ Moreover, due to the reaction kinetics between organic/inorganic halide and tin halide salts is faster than its lead analogs,^19^, ^46^, ^156^ the control of tin perovskite crystallization during the deposition process is more challenging. Therefore, developing novel preparation methods to achieve high‐quality tin perovskite film is of great importance to boost both efficiency and stability of Sn‐based PSCs. In lead‐based perovskites, the methods for morphology engineering include addition of additives,^157^, ^158^, ^159^ solvent engineering,^160^, ^161^ vacuum engineering,^162^, ^163^ thermal annealing,^164^ self‐healing,^165^ vapor deposition,^166^ and so on. Herein, the reported methods used for preparing high‐quality tin perovskite layers are introduced.

##### Additives for the Morphology Control

**Figure 10:**
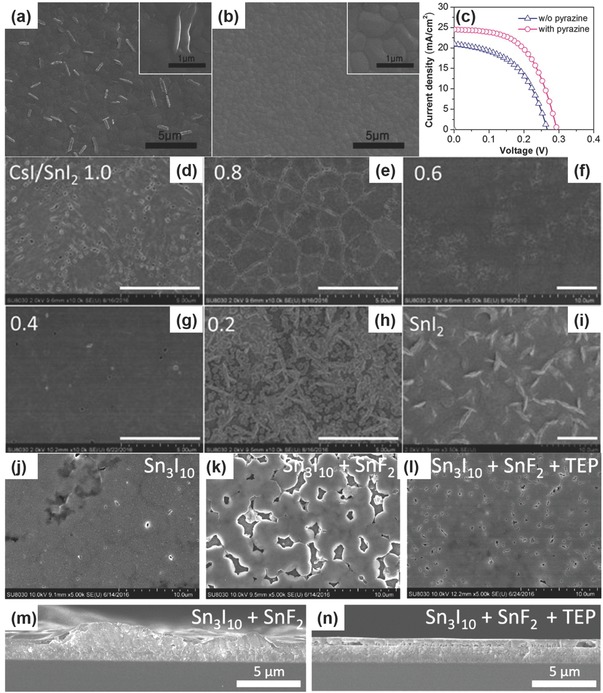
SEM images a) in the absence of pyrazine and b) in the presence of pyrazine and c) *J–V* curves of FASnI_3_ perovskite film in the presence of SnF_2_ with and without pyrazine. Reproduced with permission.^58^ Copyright 2016, ACS. d–i) SEM images of the CsSnI_3_ perovskite films grown with various CsI/SnI_2_ molar ratios, together with a neat SnI_2_ film. Reproduced with permission.^65^ Copyright 2017, ACS. Top surface SEM images of j) pristine 2D Sn_3_I_10_ film, k) (Sn_3_I_10_ + SnF_2_) film, and l) (Sn_3_I_10_ + SnF_2_ + TEP) film. Cross‐sectional SEM images of m) (Sn_3_I_10_ + SnF_2_) film and n) (Sn_3_I_10_ + SnF_2_ + TEP) film. Reproduced with permission.^63^ Copyright 2017, ACS.

Additives such as methyl ammonium chloride (MACl),^157^ 1,8‐diiodooctane (DIO),^158^ and butyl phosphonic acid 4‐ammonium chloride (4‐ABPACl)^159^ have been successfully used to obtain high‐quality perovskite films for high‐efficiency lead‐based PSCs. In the case of tin perovskite, the popularly used SnF_2_ additive for the elimination of the Sn vacancy could result in poor film morphology and bad device performance due to the phase separation of SnF_2_ within the perovskite film.^6^, ^135^ In this regard, Lee et al.^58^ introduced pyrazine into FASnI_3_ perovskite precursors in conjunction with SnF_2_ in the form of the SnF_2_‐pyrazine complex (**Figure** **10**a,b). The complex is believed to assist a uniform distribution SnF_2_ in the perovskite film, thereby substantially improving the morphology of FASnI_3_ perovskite. Finally, the resulted FASnI_3_ PSCs achieved a maximum PCE of 4.8% with high reproducibility (Figure 10c). Besides being a reducing agent, hypophosphorous acid (HPA) can also act as a morphology controller^73^ in the fabrication process of all‐inorganic CsSnIBr_2_ mesoscopic PSCs. HPA has the P—O bond that could strongly coordinate with Sn^2+^, producing HPA‐CsSnIBr_2_ clusters via Sn—O—P—O—Sn coordination bonds.^167^ The formed clusters in precursor solution promoted the growth of perovskite crystals and expelled the redundant SnF_2_ to the surface of the film. Due to the suppressed SnF_2_ phase separation in the CsSnIBr_2_ thin films, the highest reported PCE of ≈3.2% for the all‐inorganic Sn‐based PSCs was achieved. Likewise, hydrazine^41^, ^65^ was used not only as reducing agent to reduce the oxidization of Sn^2+^ to Sn^4+^, but also as modifier asserted by Song et al.^41^ to achieve better film morphologies for enhanced device efficiencies. Unfortunately, there was no explanation of why hydrazine could do a good job in ameliorating perovskite film morphologies. Moreover, a hydrazine atmosphere can help the disperse of SnI_2_ 65 to improve the quality of perovskite film, as indicated by no observable agglomeration of SnI_2_ from the SEM/EDS characterization (Figure 10d–i). The corresponding device displayed the best PCE (4.81%) of CsSnI_3_‐based PSCs, which is in the meantime much higher than the SnF_2_ 41 modified devices (1.83%). Additionally, triethyl phosphine (TEP)^63^ as a soft Lewis base can form intermediate complexes with Sn^2+^ species via weak coordinating interaction, which could slow down the perovskite crystallization process and improve the film morphology as well as the device performance. The devices with TEP showed increased FF in average (from 42.0% to 53.7%), and an average PCE from 1.15% to 1.75%, with the champion device reaching ≈2% efficiency (Figure j–n).

##### Solvent Engineering

**Figure 11:**
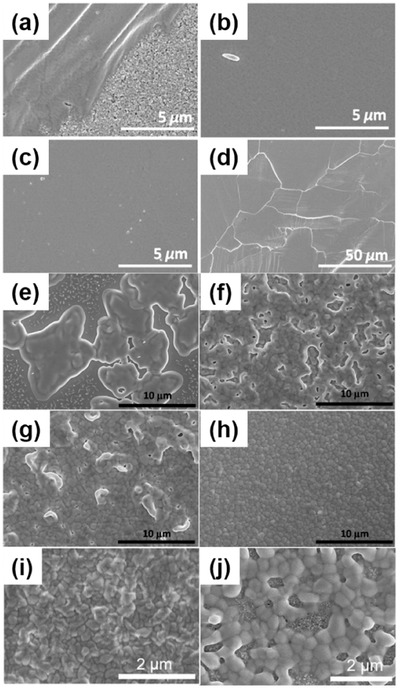
SEM images of the as‐obtained MASnI_3_ perovskite layer on mesoporous TiO_2_ from different solvents, a) DMF, b) NMP, and c,d) DMSO with different magnifications. Reproduced with permission.^47^ Copyright 2015, ACS. FASnI_3_ films deposited on PEDOT: PSS by different anti‐solvent drippings: e) no dripping, f) chlorobenzene, g) toluene, and h) diethyl ether. Reproduced with permission.^56^ SEM images of FASnI_3_ with i) diethyl ether and j) chlorobenzene as antisolvent. Reproduced with permission.^57^ Copyright 2016, ACS.

Solvent engineering technique has been proved many times as the most effective method for preparing high‐quality lead perovskite films to achieve high‐performance PSCs.^160^, ^161^, ^168^, ^169^, ^170^ The work on solvent engineering of Sn‐based perovskite was first reported by Hao et al.,^47^ where they investigated the solvent effects on the crystallization of the MASnI_3_ perovskite films. They found that highly uniform, pinhole‐free perovskite films can be obtained by using a dimethyl sulfoxide (DMSO) as solvents in perovskite precursor. And the transitional SnI_2_·3DMSO intermediate phase was very important in achieving a high‐quality perovskite film. The heterojunction depleted hole‐transporting layer‐free solar cells based on mesoporous TiO_2_ showed a high photocurrent up to 21 mA cm^−2^ and a PCE of 3.15%. Furthermore, Lee et al.^58^ used mixed solvents of *N,N*‐dimethylformamide (DMF) and DMSO in FASnI_3_ precursor solution followed by toluene drop‐casting, which led to uniform and dense FASnI_3_ perovskite layers. The role of DMSO was to retard the crystallization of FAI and SnI_2_ during spin‐coating process (**Figure** **11**a–d). The realized smooth and dense perovskite layer enables a maximum efficiency up to 4.8% for FASnI_3_‐based PSCs and the encapsulated devices kept stable for over 100 d. The same method was also applied in low‐dimensional tin halide perovskites (PEA)_2_(FA)_8_Sn_9_I_28_ to obtain compact and smooth perovskite surface morphology.^64^ Chlorobenzene,^161^ as a common antisolvent applied in lead‐based PSCs, was adopted by Zhang et al.^60^ as antisolvent to achieve a dense FASnI_2_Br film giving a device PCE of 1.72%. As an antisolvent, diethyl ether seems to work better than chlorobenzene in improving the morphology of Sn‐based perovskites.^56^, ^57^ For example, recently, Liao et al.^56^ used diethyl ether as an antisolvent in solvent engineering process to synthesize uniform and pinhole‐free FASnI_3_ perovskite thin films. The fresh spin‐coated films showed a reddish intermediate state after dripping with diethyl ether, which might be crucial for the good film morphology, in contrast to chlorobenzene and toluene‐based antisolvents, which led to black films immediately (Figure 11e–h). Champion PCE of 6.22% and high reproducibility were achieved based on FASnI_3_ PSCs, showing good stability for 30 d (maintaining 85% of its initial efficiency stored in dark and glove box) with encapsulated cells. Later, Ke et al.^57^ also used diethyl ether as antisolvent to prepare uniform FASnI_3_ perovskite films with high surface coverage on the neat TiO_2_ and TiO_2_–ZnS substrates. They pointed out that the use of diethyl ether as an antisolvent in fabrication procedure was advantageous to inhibiting phase separation caused by excess SnF_2_. They proved again that the film prepared with chlorobenzene as antisolvent exhibited poor film coverage (Figure 11i,j). However, Zhao et al.^28^ used chlorobenzene as antisolvent to obtain (FA)_0.75_(MA)_0.25_SnI_3_ and FASnI_3_ film with complete coverage and no phase separation. The exceptionally good effect of chlorobenzene may be due to the use of different solution solvent. This conjecture needs to be proved by more studies.

**Figure 12:**
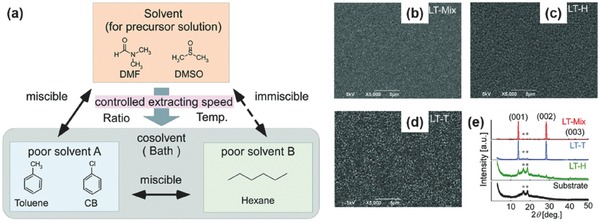
a) Concept illustration for controlling the crystallization speed using the technique of adding a cosolvent in solvent bathing; Scanning electron microscopy (SEM) images for the b) LT‐Mix, c) LT‐T, and d) LT‐H films. The X‐ray diffraction spectra for mixed solvent (red), toluene (blue), and hexane (green) are shown in (e). Reproduced with permission.^48^ Copyright 2017, RSC.

Solvent–solvent extraction technique is a derivative method of solvent engineering, which was first proposed by Zhou et al.^168^ for the fabrication of high‐quality lead perovskite thin films. The same method was applied by Milot et al.^171^ in Sn‐based perovskites with DMF and DMSO as mixed solvents for perovskite precursor solution. The wet film was immediately immersed into an antisolvent (anisole) after spin‐coating, producing a smooth, and continuous FASnI_3_ thin film. An appropriate crystallization speed is very important to the morphology of the perovskite films. Very recently, Fujihara et al.^48^ employed a mixture of toluene and hexane as the antisolvents and DMSO as the good solvent (**Figure** **12**a). Depending on the ratio of antisolvents and temperature, they can control the crystallization speed of the MASnI_3_ perovskite films to achieve high surface coverage perovskite films on a planar PEDOT: PSS electrode (Figure 12b–e). Planar junction devices with the structure of glass/ITO/PEDOT: PSS/MASnI_3_/C_60_/BCP/Ag, exhibited an efficiency of 2.14 ± 0.35% with a high open circuit voltage of 0.45 ± 0.01 V and long lifetime of over 200 h under 1 Sun illumination conditions (AM1.5, 100 mW cm^−2^).

##### Vapor Deposition

**Figure 13:**
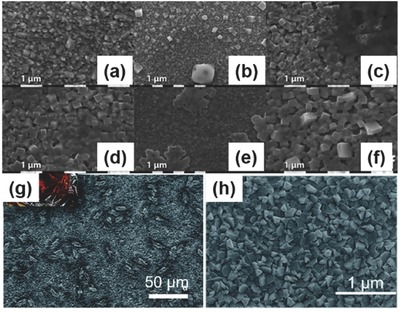
SEM images of a) 100 nm vapor‐deposited SnI_2_, nonannealed MASnI_3_ perovskite films prepared by spin‐coating of b) 6 mg mL^−1^, c) 10 mg mL^−1^, d) 20 mg mL^−1^, e) 40 mg mL^−1^ MAI solutions, and a f) MASnI_3_ perovskite film prepared by use of 20 mg mL^−1^ MAI and subsequent annealing at 80 °C for 10 min; Reproduced with permission.^173^ SEM image of coevaporated g) MASnBr_3_ (MABr:SnBr_2_ = 4:1) Reproduced with permission.^52^ Copyright 2015, RSC and h) MASnI_3_. Reproduced with permission.^50^ Copyright 2016, RSC.

Generally, vapor deposition process is known to provide higher controllability over perovskite films fabrication in terms of higher homogeneity, smoothness, and surface coverage than solution‐processed films.^166^, ^172^ Moreover, the vacuum condition adopted in vapor deposition process is especially beneficial to air‐sensitive tin perovskite. In 2015, Weiss et al.^173^ proposed a two‐step process combining vapor‐deposited SnI_2_ precursor films and solution deposited MAI for the preparation of MASnI_3_ perovskite films. The results showed homogeneous preformed SnI_2_ film and complete conversion of SnI_2_ to MASnI_3_. The final film showed complete surface coverage even with such short contact period (**Figure** **13**a–f). In the same year, smooth MASnBr_3_ thin films were synthesized via sequential evaporation by Jung et al.^52^ (Figure 13g,h). The obtained planar structure device showed an efficiency of 1.12%. In addition, SnF_2_‐doped CsSnBr_3_ film with excellent ambient air stability was also prepared by sequential vapor deposition method.^75^ Later, a hybrid thermal evaporation method at room temperature for the fabrication of high‐quality MASnI_3_ perovskite thin film was reported by Yu et al.^50^ The as‐deposited MASnI_3_ thin films have excellent morphology, with smooth surfaces, high surface coverage, and strong crystallographic preferred orientation along the <100> direction. Inverted planar architecture solar cells devices were fabricated based on these films and gave an open‐circuit voltage up to 494 mV.

##### Vapor‐Assisted Solution Process

**Figure 14:**
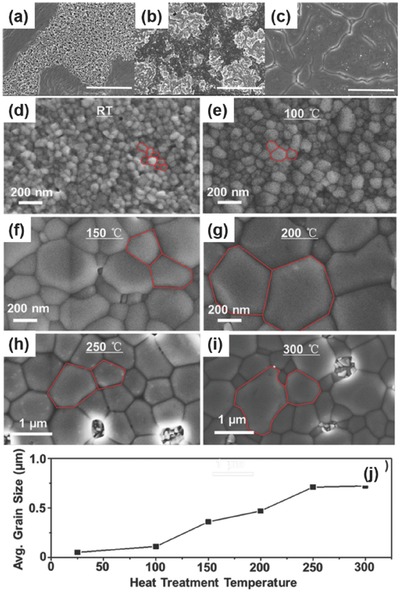
a) One‐step MASnI_3_; b) VASP–MASnI_3_; c) LT–VASP‐MASnI_3_; Reproduced with permission.^135^ Copyright 2016, ACS. SEM micrographs of the B‐g‐CsSnI_3_ thin films annealed at different temperatures for 2 min. d) As‐deposited, e) 100 °C, f) 150 °C, g) 200 °C, h) 250 °C, and i) 300 °C. j) Average grain size of the B‐g‐CsSnI_3_ thin films with increasing annealing temperature. Reproduced with permission.^67^

The so‐called vapor‐assisted solution process (VASP) was first reported to construct a high‐quality MAPbI_3_ film by Chen et al.^174^ in 2013. To improve tin perovskite surface coverage, Yokoyama et al.^135^ developed low‐temperature vapor‐assisted solution process (LT‐VASP), a kinetically control gas‐solid reaction method, to prepare lead‐free MASnI_3_ thin films. They pointed out that the substrate temperature (60−80 °C window) of preformed solid SnI_2_ is very important for achieving homogeneous and high surface‐coverage perovskite films. The acquired high‐quality MASnI_3_ films were fabricated in solar cells with an efficiency of 1.86% with good reproducibility. It is important to point out that LT‐VASP method is a pioneer work that explored alternative suitable fabrication methods for tin perovskite films (**Figure** **14**a–c).

##### Thermal Annealing

Thermal annealing is a critical step in most of the perovskite film deposition steps. It can influence the film formation of perovskites significantly by driving solid‐state coarsening of perovskite grains. An interesting work was done by Wang et al.^67^ involving an all‐inorganic and thermally stable B‐γ‐CsSnI_3_ films deposited by spin‐coating. The films were postannealed at different temperatures over a range between 100 and 300 °C for 2 min to coarsen the grains. The B‐γ−CsSnI_3_ thin films annealed at 150 °C displayed large grain size, high film smoothness, and moderate Sn vacancy (*V*_Sn_) generation, which are responsible for the best performing PSC devices. The B‐γ‐CsSnI_3_ film after 150 °C annealing was applied in an inverted planar device architecture with nickel oxide (NiO*_x_*) as the photocathode. They achieved a PCE of 3.31% without the use of any additive. This work demonstrated that proper thermal annealing is another efficient method for preparing high‐performance Sn‐based PSCs (Figure 14d–j).

#### 2.1.4. Summary

In summary, as two less toxic family members of Pb, Sn and Ge are deemed as the redeemer to the toxic Pb element and tremendous efforts have been put in optimizing the materials and devices. So far, Sn‐based perovskites with lower bandgaps than lead analogs, have obtained a “stable” efficiency up to 6%. In this case, the issue of Sn^2+^ oxidization has been partly overcome by adding divalent tin halide additives and some reductive reagents. Additionally, the poor morphology of the tin halide perovskite layer has been improved by the various fabrication methods. Recently, great progress has been made in Sn‐based PSCs with inverted device architecture,^28^, ^56^, ^64^, ^66^, ^67^ due to the omission of doped HTMs. Unlike Pb‐based PSCs where the high efficiencies are usually achieved with doped HTMs, the use of dopants will accelerate the deterioration of tin perovskites. Hence, exploring high‐performance and dopant‐free HTMs is very important to get efficient and stable Sn‐based PSCs. And it is necessary to further study tin‐based perovskite material fundamentally (such as the mechanisms of self‐doping) and explore novel device structures with different charge selective contact materials toward more efficient and stable Sn‐PSCs. For germanium perovskites, the study of these compounds is very rare in the photovoltaic application so far. In this case, due to the relatively small ionic radius of Ge^2+^, the octahedra [GeX_6_]^3−^ network is heavily distorted, which leads to wide band gap (>1.6 eV). In addition, the poor solubility of these compounds in polar solvents causes terrible morphology with low efficiency of only 0.2% from solution process. It is urgent to find new preparation methods of germanium perovskites for more efficient Ge‐based PSCs. Moreover, the easy oxidation of Ge^2+^ and Sn^2+^ will be definitely a challenge.

### 2.2 The Group 15 Metals Bi and Sb‐Based Absorbers

Beyond group 14 elements, two of group 15 metals in the periodic table, bismuth (Bi) and antimony (Sb) have been also studied for replacing lead in the solar energy absorbing materials. In this section, a brief introduction of typical A_3_Bi_2_X_9_, ABi_3_X_10_, A_3_Sb_2_X_9_ (A = MA+, FA+, Cs+; X = I^−^, Br^−^, Cl^−^) polymorphs and other derivatives will be given. Compared with Sn‐based perovskites, they form more diverse dimensionality in terms of the connection type of BiX_6_^3−^ (SbX_6_^3−^) octahedron.^101^ Here we divided them into 0D, 1D, 2D, and 3D structures.

#### 2.2.1 Bismuth‐Based Absorbers

Being adjacent to Pb^2+^ in the periodic table, Bi^3+^ has the similar 6s^2^6p^0^ electronic configuration, which endows MAPbX_3_ with the strong light absorption and long carrier lifetimes.^176^ More importantly, it is much less toxic than Pb,^177^, ^178^ and has been used in organic synthesis and medicines.^179^, ^180^, ^181^ Hence, Bi‐based perovskite or hybrid materials are attractive options to replace lead perovskites.

##### 2.2.1.1. 0D Hybrid Materials:

**Figure 15:**
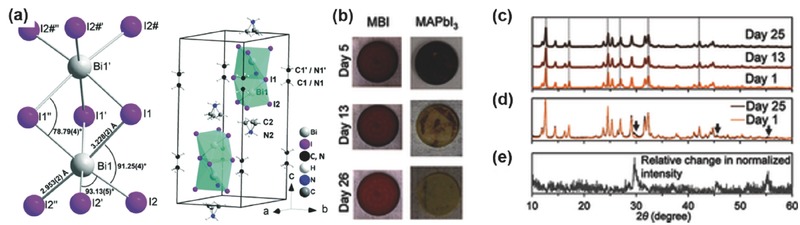
a) Crystal structure of (CH_3_NH_3_)_3_Bi_2_I_9_ (MBI) (left) local structure of the Bi_2_I_9_^3−^ anion and (right) cation and anion positions in the unit cell. Reproduced with permission.^183^ Copyright 2016, RSC. Air stability of MBI, measured from mid‐July to mid‐August in Cambridge, MA, USA. b) Photographs of MBI and MAPbI_3_ on quartz over time in the ambient air. c,d) Normalized XRD patterns of MBI over time with air exposure. e) The relative change in the normalized intensity of the diffraction peaks of MBI (day 25 vs. day 1). Reproduced with permission.^92^

*MA_3_Bi_2_I_9_ Hybrid Bismuth Iodides*: Among all the reported bismuth‐based absorbers, organic–inorganic hybrid bismuth halide MA_3_Bi_2_I_9_ is the most studied polymorph type. Owing to the tervalence state of Bi^3+^, the solid structure of MA_3_Bi_2_I_9_ features two face‐sharing 0D perovskite structure,^88^, ^182^, ^183^ which is constructed by the MA^+^ surrounded binuclear octahedral (Bi_2_I_9_)^3−^ clusters, contrasting to the 3D MAPbI_3_ perovskite (**Figure** **15**a).

The single crystal of MA_3_Bi_2_I_9_ normally displays color like red wine^89^ and has regular hexagonal shape with a diameter ranging from 100 to 200 µm,^87^ or even up to 4–5 mm,^89^ adopting *P*6_3_/*mmc* space group.^88^, ^182^, ^184^ The optical bandgap of MA_3_Bi_2_I_9_ was reported to be ≈1.94–2.26 eV^87^, ^88^, ^89^ with absorption coefficient up to ≈1 × 10^5^ cm^−1^, the same order of magnitude with MAPbI_3_.^185^ The valence band maximum (VBM) of the MA_3_Bi_2_I_9_ was measured to be 5.63 eV by photoelectron spectroscopy in the air (PESA)^89^ and 5.9–6.0 eV by ultraviolet photoelectron spectroscopy (UPS)^87^, ^89^ in vacuum, respectively, which are all well aligned with the conduction band of TiO_2_. The electron mobility of MA_3_Bi_2_I_9_ single crystal is 29.7 cm^2^ V^−1^ s^−1^ as estimated by the space charge limited conduction (SCLC),^89^ which is comparable to that of MAPbI_3_ (38 cm^2^ V^−1^ s^−1^).^186^ The same carrier mobility was estimated to be 1 cm^2^ V^−1^ s^−1^ by Hall Effect.^87^ The phase‐pure and compact MA_3_Bi_2_I_9_ film showed long PL decay over 0.76 ns, with the bulk lifetime approach to 5.6 ns. And this film exhibited robust air stability than MAPbI_3_ after 25 d of continuous air exposure with 61% relative humidity (Figure 15b,c). It was speculated that the high air stability is owing to the formation of Bi_2_O_3_ or BiOI from BiI_3_, which could serve as a protective layer to prohibit the ingress of water and oxygen into bulk materials.^92^ Therefore, on account of its good optoelectronic properties and excellent stability, MA_3_Bi_2_I_9_ is the prevailing candidate materials for replacing lead perovskites in the photovoltaic application.

**Figure 16:**
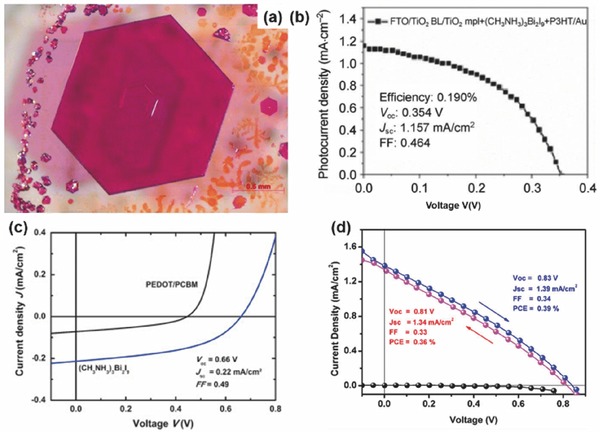
a) Large 70 mm thick single crystal of (CH_3_NH_3_)_3_Bi_2_I_9_ grown on the ITO substrate. Reproduced with permission.^89^ Copyright 2016, RSC. *J*–*V* curve of b) the P3HT based n–i–p device. Reproduced with permission.^87^ Copyright 2016, Springer. And c,d) p–i–n devices. c) Reproduced with permission.^91^ Copyright 2016, Elsevier. d) Reproduced with permission.^83^ Copyright 2017, ACS.

The first report on MA_3_Bi_2_I_9_ as light absorbers used in solar cells was by Park et al.^88^ They studied the morphology of MA_3_Bi_2_I_9_ and Cl‐doped (MA_3_Bi_2_I_9_Cl*_x_*) films on TiO_2_ substrate by SEM. The results showed that MA_3_Bi_2_I_9_ had interconnected layers compared to the more particle‐like structure of MA_3_Bi_2_I_9_Cl*_x_*. Meanwhile, they pointed out that the excellent surface morphology of MA_3_Bi_2_I_9_ could be propitious to form excitons with lower energy. In traditional mesoscopic solar cells, the best device based on MA_3_Bi_2_I_9_ showed *J*_SC_ = 0.52 mA cm^−2^, *V*_OC_ = 0.68 V, FF = 0.33, and η = 0.12%. By contrast, MA_3_Bi_2_I_9_Cl*_x_* showed *J*_SC_ = 0.18 mA cm^−2^, *V*_OC_ = 0.04 V, FF = 0.38, and η = 0.003%. The extreme low *V*_OC_ of MA_3_Bi_2_I_9_Cl*_x_* was attributed to the poor morphology with perovskite particles surrounded by amorphous BiCl_3_. Two months later, Lyu et al.^87^ employed poly(3‐hexylthiophene‐2,5‐diyl) (P3HT) as the HTM to replace spiro‐OMeTAD in the MA_3_Bi_2_I_9_‐based solar cells with a mesoscopic structure, which showed a PCE of 0.19% (**Figure** **16**b).

It became a comment sense that the performance of MA_3_Bi_2_I_9_‐based PSCs was improving due to the better morphology of the absorber layer. Singh et al.^86^ deposited uniform MA_3_Bi_2_I_9_ layers atop mesoporous anatase TiO_2_ and exhibited best PCE of 0.2%, as well as 10 weeks stability of the device in ambient condition. Zhang et al.^187^ used smooth indium tin oxide (ITO)/glass substrate to achieve a dense MA_3_Bi_2_I_9_ thin film, which gave a maximum PCE of 0.42% and high FF up to 0.64. This is one of the highest performances among MA_3_Bi_2_I_9_‐based lead‐free PSCs.

More efforts have been paid to modulate the morphology of MA_3_Bi_2_I_9_ perovskite. For example, 1‐methyl‐2‐pyrrolidinone (NMP)^85^ as morphology controller was added into the MA_3_Bi_2_I_9_–DMF precursor solution, producing a homogeneous film of MA_3_Bi_2_I_9_. The device yielded highly reproducible PCE of 0.31% and kept stable for 30 d in an ambient atmosphere (relative humidity of 50–60%). On the other hand, antisolvent assisted crystallization (ASAC) method also was used to improve MA_3_Bi_2_I_9_ thin films like the cases in the lead‐ and tin‐based PSCs. Abulikemu et al.^89^ first used this method, but gave a PCE of only 0.11%. Very recently, Mali et al.^84^ achieved cuboid‐shaped crystals of MA_3_Bi_2_I_9_ on the surface of mesoporous TiO_2_ by this method. The obtained thin film had excellent air stability with almost no color change even after two month exposure to air. The best solar cell devices made from this kind of films exhibited a PCE of 0.36%, with no substantial efficiency loss after 60 d. Gas‐assisted deposition method was first reported by Huang et al.^188^ to create uniform and dense lead perovskite thin films. Naturally, this method was used^90^ to improve the quality of MA_3_Bi_2_I_9_ films. Ultimately a PCE of merely 0.08% was obtained, which is 17% higher compared with the conventional one‐step method. Besides the common n–i–p‐type devices, the first p–i–n planar heterojunction device of MA_3_Bi_2_I_9_ perovskite was reported by Öz et al.^91^ with PCE of ≈0.1%. To achieve a smooth, uniform, and compact MA_3_Bi_2_I_9_ film in the p–i–n device structure, Ran et al.^83^ reported a two‐step (evaporation and spin‐coating) process of MA_3_Bi_2_I_9_ and obtained a PCE of 0.39% and *V*_OC_ as high as 0.83 V, which is the highest *V*_OC_ among MA_3_Bi_2_I_9_‐based solar cells so far (Figure 16d).

**Figure 17:**
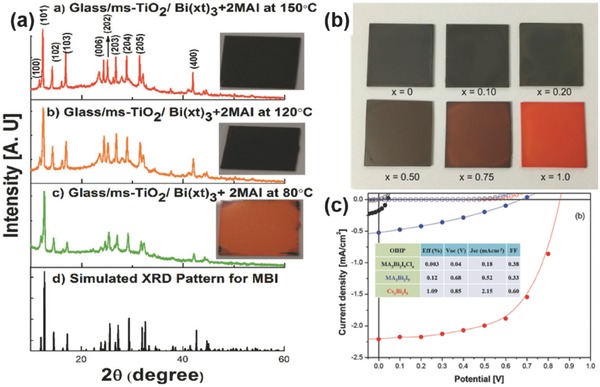
a) XRD patterns and color for sulfur‐doped MBI at different postheating temperatures. Reproduced with permission.^93^ Copyright 2016, ACS. b) Color of (BiI_3_)_1−_ *_x_*(MBI)*_x_* films prepared by the solution method. Reproduced with permission.^94^ Copyright 2017, Elsevier. c) *J*–*V* curves MA_3_BiI_2_I_9_Cl*_x_*, MA_3_Bi_2_I_9_, and Cs_3_Bi_2_I_9_‐based solar cell devices. Reproduced with permission.^88^

*Sulfur‐Doped MA_3_Bi_2_I_9_*: Sulfur‐doped MA_3_Bi_2_I_9_ was developed^93^ to reduce bandgap of MA_3_Bi_2_I_9_ (2.1 eV), which is a relatively higher for the ideal single junction solar cell.^129^ Sulfur‐doped bismuth perovskites were obtained by in situ sulfur doping of MA_3_Bi_2_I_9_ through the thermal decomposition of Bi(xt)_3_ (xt = ethyl xanthate) precursor. The color of obtained perovskite films changed from orange to black when annealed from 80 to 150 °C, and there was a notably red shift in the optical absorption edge (**Figure** **17**a). The bandgap of sulfur‐doped bismuth perovskite was measured to be 1.45 eV, which is even lower than the prototype MAPbI_3_.^189^ Moreover, sulfur‐doped MA_3_Bi_2_I_9_ exhibited a high carrier mobility of 2.28 cm^2^ V^−1^ s^−1^, about twice than that of pristine MA_3_Bi_2_I_9_. This work showed that doping could reduce the bandgap of MA_3_Bi_2_I_9_ while improving the charge transport, and further might enhance the performance of MA_3_Bi_2_I_9_‐based solar cells. However, unfortunately, there was no device prepared based on such kinds of material.

*(BiI_3_)_0.8_(MA_3_Bi_2_I_9_)_0.2_*: One of the problems with MA_3_Bi_2_I_9_ is that its bandgap is even wider than that of BiI_3_ (1.8 eV). Therefore, to improve light absorption of MA_3_Bi_2_I_9_, Lan et al.^94^ designed active composite layers taking advantages of optoelectronic properties of BiI_3_ 190, 191, 192, 193 and suitable energy level alignment of MA_3_Bi_2_I_9_ with TiO_2_ (Figure 17b). When 20% of MA_3_Bi_2_I_9_ perovskite was introduced into the active layers, the (BiI_3_)_0.8_(MA_3_Bi_2_I_9_)_0.2_ solar cells displayed improved *V*_OC_ from 0.44 to 0.57 V, with a PCE of 0.08%.

*A_3_Bi_2_I_9_ (A: Cesium, Formamidinium, Imidazolium, Cyclohexyl Ammonium)*: Replacing MA with formamidinium (FA), the FA_3_Bi_2_I_9_ 95 exhibits the same structure as MA_3_Bi_2_I_9_, with a bandgap of 2.0 eV. Additionally, when more bulky cations like imidazolium and cyclohexyl ammonium are used as the A cation, 0D perovskite‐like structure are formed. The synthesized (C_3_H_5_N_2_)_3_[Bi_2_I_9_]^96^ has two temperature induced solid–solid structural phase transition, and (C_6_H_14_N)_3_Bi_2_I_9_ 97 has red emissions at room temperature. All‐inorganic bismuth halide compounds have also been studied to replace lead perovskites in PSCs. Cs_3_Bi_2_I_9_ as a light harvester was first studied by Park et al.^88^ with face‐sharing octahedra dimer ((Bi_2_I_9_)^3−^, P6_3_/mmc space group, 0D structure) similar to MA_3_Bi_2_I_9_. It possesses a bandgap of 2.2 eV close to MA_3_Bi_2_I_9_ (2.1 eV). A detailed study of its band gap structure was shown by Zhang et al.^194^ Compared with MA_3_Bi_2_I_9_ and MA_3_Bi_2_I_9_Cl*_x_*, Cs_3_Bi_2_I_9_ displays a relatively high PL yield, suggesting low losses in nonradiative recombination. Consequently, the best Cs_3_Bi_2_I_9_‐based solar cell shows a PCE of 1.09% (*V*_OC_ = 0.85 V, *J*_SC_ = 2.15 mA cm^−2^, and FF = 0.6) (Figure 17c).

##### 2.2.1.2. 1D Hybrid Materials:

*MA_3_Bi_2_Cl_9_*: MA_3_Bi_2_Cl_9_ 98 is a 1D organic–inorganic hybrid bismuth compound with zig‐zag double chains of distorted BiCl_6_^3−^‐octahedra structure. The past studies of MA_3_Bi_2_Cl_9_ focused mainly on structural phase transition, which revealed two‐phase transitions^195^ at *T*_c1_ = 349 K and *T*_c2_ = 247 K, respectively.

**Figure 18:**
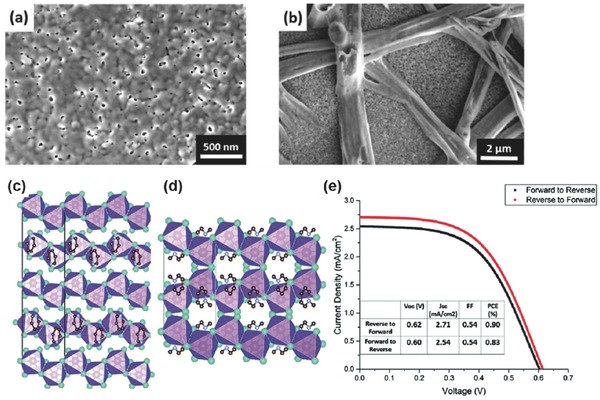
Top‐view SEM image of a) HDABiI_5_ and b) MAPbI_3_ deposited on mTiO_2_/cTiO_2_/FTO. Reproduced with permission.^198^ Copyright 2016, RSC. Crystallographic packing diagrams of c) [py][BiI_4_] and d) [mepy][BiI_4_]; e) Boltzmann‐fitted *J*–*V* curves of the [py][BiI_4_] champion cell. Reproduced with permission.^37^ Copyright 2017, RSC.

*(H_3_NC_6_H_12_NH_3_)BiI_5_ and (TMP)BiX_5_*: 1,6‐hexane diammonium bismuth halide (H_3_NC_6_H_12_NH_3_)BiI_5_ (HDABiI_5_) showing 1D chains structure has early been reported by Mousdis et al.^196^ and Mitzi et al.,^197^ respectively (**Figure** **18**a,b). Owning an optical bandgap of ≈2.0 eV, Fabian and Ardo^198^ first applied HDABiI_5_ as a light absorber in PSCs. HDABiI_5_ layer had near‐complete surface coverage on mesoporous TiO_2_. The low volatility of organic 1,6‐hexane diamine group endowed HDABiI_5_ with good thermal stability up to 200 °C. However, the final PCE of the device was only 0.027%. TMP (*N,N,N′,N′*‐tetramethylpiperazine) can also be used as organic cation and yielded (TMP)BiX_5_ (X = Cl, Br, I) with 1D chains structure and optical band gaps of 2.02–3.21 eV.^101^

*C_5_H_6_NBiI_4_ and C_6_H_8_NBiI_4_*: Another two 1D chained organic–inorganic iodobismuthates, C_5_H_6_NBiI_4_ ([py][BiI_4_]) (py: pyridinium) and C_6_H_8_NBiI_4_ ([mepy][BiI_4_]), (mepy: methyl pyridinium) were prepared by Li et al.^37^ and applied in HTM‐free PSCs with mesoscopic anode and carbon counter electrode (Figure 18c,d). They pointed out that the protonated aromatic heterocycles play an active role in intermolecular interactions through the frontier orbitals, which endows them with pseudo‐3D charge transfer ability. [py][BiI_4_] and [mepy][BiI_4_] have bandgap of 1.98 and 2.17 eV, respectively. The best device efficiency of 0.9% was obtained using [py][BiI_4_], which is comparable with that of other reported Bi–iodide based devices^83^, ^88^, ^187^ (Figure 18e).

##### 2.2.1.3. 2D Hybrid Materials:

**Figure 19:**
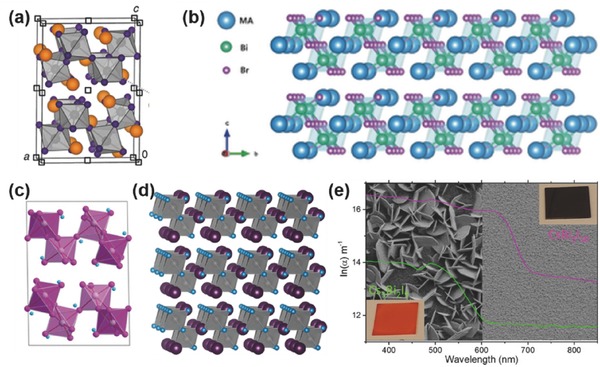
2D layered crystal structures of a) Rb_3_Bi_2_I_9_ (*P*2_1_/*n*); Reproduced with permission.^36^ Copyright 2015, ACS. b) MA_3_Bi_2_Br_9_; Reproduced with permission.^102^ c) (NH_4_)_3_Bi_2_l_9_; Reproduced with permission.^103^ Copyright 2016, American Institute of Physics. d) Cs_3_Bi_2_Br_9_; Reproduced with permission.^199^ Copyright 2017, ACS. e) Photo and SEM images of Cs_3_Bi_2_I_9_ (left) and CsBi_3_I_10_ (right). Reproduced with permission.^100^ Copyright 2016, ACS.

*A_3_Bi_2_I_9_ (A: K^+^, Rb^+^, NH_4_^+^)*: By replacing the Cs^+^ in typical 0D Cs_3_Bi_2_I_9_ perovskite with K^+^ and Rb^+^, K_3_Bi_2_I_9_ and Rb_3_Bi_2_I_9_ 36 are achieved. The decreased size of cations induces 2D layered defect‐perovskite structure, with corrugated layers of Bi–I octahedra. K_3_Bi_2_I_9_ and Rb_3_Bi_2_I_9_ have direct bandgaps of 2.1 eV, while Cs_3_Bi_2_I_9_ has an indirect bandgap of 1.9 eV (**Figure** **19**a). Lehner et al. pointed out that direct gaps coupled with the high DOS with a strong p‐character across the gap are criteria for effective light absorption. Besides, substituting MA cation for NH_4_^+^ cation in MA_3_Bi_2_I_9_, Sun et al.^103^ achieved a 2D layered perovskite‐like architecture (NH_4_)_3_Bi_2_I_9_, with a dark red color and a bandgap of 2.04 eV. But no device study was provided in these reports (Figure 19c).

*A_3_Bi_2_Br_9_ (A: MA, Cs)*: Besides the change of A cations, when the halide atom is changed to Br^−^, one can change the typical 0D Cs_3_Bi_2_I_9_ to 2D layered perovskites. For example, MA_3_Bi_2_Br_9_ 102 crystallizes into trigonal symmetry ($P\bar{3}$ *m*1) space group, forming corrugated layers of BX_6_ octahedra (Figure 19b). MA_3_Bi_2_Br_9_ possesses a direct bandgap of 2.5 eV^102^ and emits at 430 nm as quantum dots with photoluminescence quantum yield (PLQY) up to 12%. Another layered inorganic halide bismuth compound, Cs_3_Bi_2_Br_9_ was reported by Bass et al.^199^ It occupies the 2D structure, with corrugated layers of corner‐sharing BiBr_6_^3−^ octahedra, as illustrated in Figure 19d, different from iodine analogs with 0D structure. Cs_3_Bi_2_Br_9_ has a large exciton binding energy of 940 meV, which is indicative of a strongly localized character and resulted in highly structured emission. However, this large exciton binding energy will extremely limit its application in photovoltaic technologies, particularly as a light harvester.

*CsBi_3_I_10_*: Johansson et al.^100^ reported another type of cesium bismuth iodine compound CsBi_3_I_10_. In contrast to previously reported Cs_3_Bi_2_I_9_, CsBi_3_I_10_ has a different orientation of crystal growth, which may explain a more uniform and smoother coverage on TiO_2_. CsBi_3_I_10_ film possesses a smaller bandgap of 1.77 eV and higher absorption coefficients up to 1.4 × 10^5^ cm^−1^, which are advantageous to PV application, compared with the bandgap of Cs_3_Bi_2_I_9_ at 2.03 eV and absorption coefficients of 7 × 10^4^ cm^−1^ (Figure 19e). For the same reason, this material was also used in a red‐light photodetector recently.^200^ The PV device with a structure of glass/FTO/compact TiO_2_/mesoporous TiO_2_/CsBi_3_I_10_/P3HT/Ag, showed a PCE of 0.4%, with a notable *J*_SC_ of 3.4 mA cm^−2^. This work proved the possibility to further increase light absorption and photocurrents in bismuth halide absorbers based solar cells.

*(TMP)_1.5_[Bi_2_I_7_Cl_2_]*: It is interesting that (TMP)[Bi_2_I_5_] with one kind of halide species show 1D chain structure, while the addition of Cl leads to the formation of (TMP)_1.5_[Bi_2_I_7_Cl_2_] with a 2D structure.^101^ It has an optical bandgap of 2.10 eV and improved electrical conductivity of 2.37 × 10^−6^ S cm^−1^. Moreover, it displayed efficient photoconductivity response and very high stability either in humid air or long‐time irradiation in a simple device.

###### 2.2.1.4. 3D Hybrid Materials (Double Perovskites):

The organic–inorganic hybrid bismuth perovskites mentioned above are all low dimensional structures with PSCs efficiency of only ≈1%. To realize the 3D perovskite architecture which has demonstrated advantages for high efficiency in lead perovskites, double perovskite with 3D structure was developed. Incorporating a monovalent metal into bismuth perovskites could yield a 3D double perovskite with the chemical formula of A^I^_2_B^I^Bi^III^X_6_. Double perovskites usually exhibits a high tolerance to defects owing to the strong ionic nature of the constituents and 3D structure similar to organolead halide perovskites.^201^ But they usually have large bandgaps that prevent absorption of the whole solar spectrum.^38^, ^109^, ^202^, ^203^

**Figure 20:**
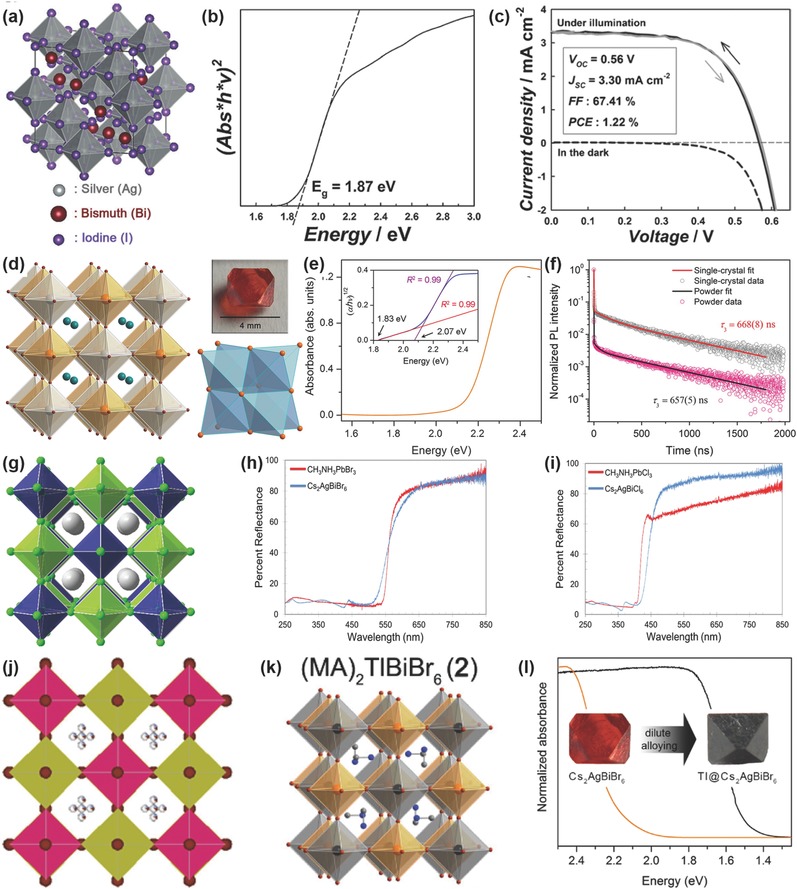
a) Single‐crystal structure of AgBi_2_I_7_ cubic structure six‐coordinated silver‐iodide octahedron sites. b) Tauc plot of AgBi_2_I_7_ from the UV/Vis spectroscopy to determine *E*_g_ under the assumption of a direct bandgap. c)  *J–V* curves in the dark and illumination under 100 mW cm^−2^ AM 1.5 G. Reproduced with permission.^114^ d) Single‐crystal structure of Cs_2_AgBiBr_6_; Photograph of the single crystal; The Bi^3+^ face‐centered‐cubic sublattice, consisting of edge‐sharing tetrahedra. e) Absorbance spectrum of Cs_2_AgBiBr_6_ powder; f) Time‐resolved room‐temperature PL and fits for the PL decay time (*t*) in powder and single‐crystal samples. Reproduced with permission.^38^ Copyright 2016, ACS. g) Refined crystal structure of Cs_2_AgBiCl_6_; Diffuse reflectance spectra for h) Cs_2_AgBiBr_6_ and CH_3_NH_3_PbBr_3_ and i) Cs_2_AgBiCl_6_ and CH_3_NH_3_PbCl_3_; Reproduced with permission.^109^ Copyright 2016, Springer.Crystal structure of (j) (MA)_2_AgBiBr_6_; Reproduced with permission.^106^ Copyright 2017, Springer. And k) (MA)_2_TlBiBr_6_; l) Photographs of Cs_2_AgBiBr_6_ and Cs_2_(Ag_1−_ *_a_*Bi_1−_ *_b_*)Tl*_x_*Br_6_ (*x* = *a* + *b* = 0.075) single crystals and change of absorption onset. Reproduced with permission.^111^ Copyright 2017, ACS.

*MA_2_B^I^Bi^III^X_6_ (X: I, Br, Cl; B: Tl, K, Ag)*: Early in 2015, Giorgi et al.^175^ proposed double‐perovskite structure MA_2_TlBiI_6_ computationally, by substituting Pb^2+^ with Tl^+^ and Bi^3+^ from parental MAPbI_3_, but stayed in theory because monovalent metal Tl is very toxic. As a compromise, the toxic thallium can be replaced by other monovalent metals like potassium. Therefore, organic–inorganic hybrid double‐perovskite MA_2_KBiCl_6_ was first synthesized by Wei et al.,^104^ which is solution processable but with a bandgap of 3.04 eV similar to that of the prototypical MAPbCl_3_ perovskite. Because its bandgap is too large to be used for the photovoltaic application. They^105^ finally turned to MA_2_TlBiBr_6_, which is isoelectronic with MAPbBr_3_. MA_2_TlBiBr_6_ adopts a space group of $Fm\bar{3}$ m like Cs_2_AgBiX_6_ (X = Cl, Br) (vide infra) and possesses a narrower bandgap of 2.16 eV than MA_2_KBiCl_6_ (**Figure** **20**k). Very recently,^106^ they synthesized another new hybrid perovskite: MA_2_AgBiBr_6_ with a low bandgap of 2.02 eV and without toxic element. MA_2_AgBiBr_6_ also forms in cubic space group $Fm\bar{3}$m, with better thermal stability (decomposition temperature up to 550 K) than MAPbBr_3_, and no obvious phase transition was detected from 120 to 360 K. Additionally, the crystal color changes from red to yellowish brown upon cooling (Figure 20j).

*Cs_2_A^I^B^III^X_6_ (A: Ag, (Ag_1−a_Tl_x_); B: Bi, In, Bi_1−x_In_x_, Bi_1−x_Sb_x_; X: Cl, Br)*: Like organic–inorganic hybrid bismuth compounds, inorganic ternary metal halides (A_3_Bi_2_X_9_) usually occupy low dimensional structures. However, before the report of organic–inorganic hybrid bismuth double‐perovskites, all‐inorganic halide bismuth double‐perovskite (3D) Cs_2_AgBiBr_6_ was already synthesized by Slavney et al.^38^ Cs_2_AgBiBr_6_ has an indirect bandgap of 1.95 eV, and the material shows a long PL decay of 660 ns at room temperature (Figure 20d–f). Additionally, Cs_2_AgBiBr_6_ shows higher stability with heat and moisture than MAPbI_3_. Almost the same time, McClure et al.^109^ reported Cs_2_AgBiBr_6_ with the chloride analogue Cs_2_AgBiCl_6_, with indirect bandgap of 2.19 and 2.77 eV, respectively. Both compounds adopt the cubic double‐perovskite structure with space group $Fm\bar{3}$ *m*. They are stable when exposed to air, but with the additional light, Cs_2_AgBiBr_6_ degrades over a period of weeks. In contrast, Cs_2_AgBiCl_6_ shows better stability with no apparent change observed. Volonakis et al.^202^ also designed and synthesized Cs_2_AgBiCl_6_ (Figure 20g–i). The bandgaps of double perovskites were between 1.95 and 3.04 eV, which were too wide to be used as absorbers in single junction photovoltaic cells. To lower bandgap, Slavney et al.^111^ incorporated Tl^+^ as a dilute impurity into Cs_2_AgBiBr_6_, achieving an opaque black octahedral perovskite crystals Cs_2_(Ag_1−_ *_a_*Bi_1−_ *_b_*)Tl*_x_*Br_6_ (0.003 < *x* = *a* + *b* < 0.075) with very stable structure. The Tl‐doped compounds Cs_2_(Ag_1−_ *_a_*Bi_1−_ *_b_*)Tl*_x_*Br_6_ displayed low bandgap down to 1.40 eV (indirect) and 1.57 eV (direct) when *x* = 0.075, which is competitive with that of MAPbI_3_.^16^ Moreover, time‐resolved photoconductivity measurements showed that the Tl‐doped materials had long‐lived carriers up to microsecond, though shorter than that of Cs_2_AgBiBr_6_ due to the extra doping of Tl. This study demonstrated the first double perovskite that has comparable band gap and carrier lifetime to those of MAPbI_3_, but regrettably, there was still toxic Tl in the compounds (Figure 20l). Additionally, through alloying of trivalence In^III^/Sb^III^ into Cs_2_AgBiBr_6_, the bandgap of double perovskite Cs_2_Ag(Bi_1−_ *_x_*M*_x_*)Br_6_ 112 (M = In or Sb) can be modulated. For example, when M is Sb^III^ and *x* = 37.5%, Cs_2_Ag(Bi_0.625_Sb_0.375_)Br_6_ had a bandgap of 1.86 eV, which is 0.41 eV lower than the previous ternary compound.

To obtain an absorber with a direct bandgap, Volonakis et al.^113^ replaced Bi with In and calculated the band structure of Cs_2_AgInX_6_ (X = Cl, Br, I) by first‐principles calculations. The combined experiments identified that Cs_2_InAgCl_6_ has a direct bandgap of 3.3 eV. The potential of A_2_B′B″X_6_ type double perovskites for PV application was further studied by theoretical methods,^105^, ^202^, ^204^ and focus was put on A_2_In^+^Bi^3+^X_6_ perovskites. For example, Zhao et al. proposed Cs_2_InBiCl_6_ and Cs_2_InSbCl_6_ with low direct bandgaps ≈1 eV by HSE+SOC calculation.^204^ However, Xiao et al.^205^ used a combination of theoretical and experimental study to show that Cs_2_InBiCl_6_ and Cs_2_InSbCl_6_ were unstable due to spontaneous oxidation of In^+^ into In^3+^. Lately, Volonakis et al.^206^ proposed the rule of designing a useful double perovskite material: mimicking the electronic structure of MAPbI_3_. To stabilize the double perovskite structure of A_2_In^+^Bi^3+^X_6_, they suggested the use of mixed‐A‐site‐cation double perovskite (Cs/MA/FA)_2_InBiBr_6_ rather than all‐inorganic double perovskites. Although their attempts to synthesize MA_2_InBiBr_6_ and FA_2_InBiBr_6_ were failed, more efforts are needed to explore the suitable composition of the cations.

Among the reported double‐perovskites, Cs_2_AgBiBr_6_ is the only one that was applied in a working device. Very recently, Greul et al.^110^ prepared phase pure Cs_2_AgBiBr_6_ films with a optimal post‐annealing temperature of 285 °C. The corresponding mesoscopic devices displayed an incredible maximum PCE of 2.43%, with JSC = 3.93 mA cm^‐2^, V_OC_ = 0.98 V and FF = 0.63. Moreover, stability of Cs_2_AgBiBr_6_‐based devices was tested under constant illumination at ambient conditions during 100 min. This work suggested the potential of double‐perovskites as lead‐free alternatives to MAPbI_3_.

*AgBi_2_I_7_ and Ag_2_BiI_5_*: Besides double‐perovskites, Kim et al.^114^ synthesized AgBi_2_I_7_ with cubic‐phases composed of vacancy‐free corner‐sharing bismuth iodide hexahedra and silver iodide octahedra. (Figure 20a–c) They fabricated dense, smooth, and pinhole‐free AgBi_2_I_7_ thin films with 200–800 nm large grains after annealing at 150 °C. The AgBi_2_I_7_ film absorbs light across the range from 350 to 750 nm, with an *E*_g_ value of 1.87 eV. They applied it in solar cells and the best AgBi_2_I_7_‐based device had a PCE of 1.22 % and showed good stability with only 8% PCE reduction over 10 d under ambient conditions. From solutions with different ratios of AgI and BiI_3_ (AgI/BiI_3_ = 2:1), Zhu et al. got a new crystal structure of Ag_2_BiI_5_ with a space group of $R\bar{3}$ m.^115^ The Ag_2_BiI_5_‐based devices showed a maximum IPCE of 45% and a promising PCE above 2%. The results show the potential of finding new lead‐free absorbers and the possibility to tune the properties of bismuth halides by adding a different ratio of precursors.

#### 2.2.2. Antimony‐Based absorbers

Antimony (Sb) is on the top right‐hand corner of lead in the periodic table, and its trivalent cation possesses a similar electronic configuration with divalent Pb^2+^. Antimonial compounds have been studied and used as therapeutic agents for human leishmaniasis and demonstrated low toxicity with appropriate regulations.^207^, ^208^ Hence, Sb is expected to be a nontoxic alternative to lead as well. Due to the high oxidation state (+3), Sb^3+^‐based halides have crystal structures of low dimensionality with the typical chemical structure A_3_Sb_2_X_9_, forming in dimer structure or layered structures.^119^, ^209^

**Figure 21:**
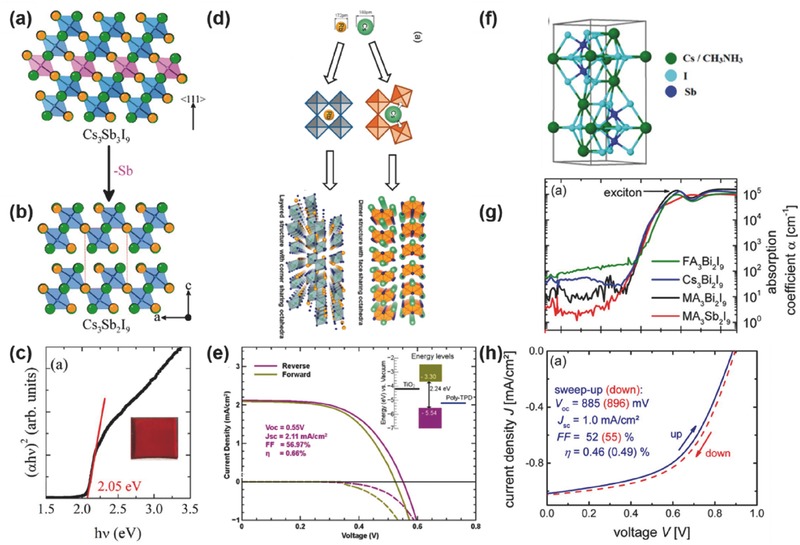
a) Removal of every third Sb layer along the 〈111〉 direction of a) the perovskite structure results in b) the 2D layered modification of Cs_3_Sb_2_I_9_; c) Bandgap of the layered modification of Cs_3_Sb_2_I_9_ (inset shows a thin film) using the Tauc relation. Reproduced with permission.^119^ Copyright 2015, Springer. d) Schematic showing the influence of A cation size on the structure of A_3_Sb_2_I_9_; e) *J*–*V* curve under forward and reverse scans of the best device with the energy levels of Rb_3_Sb_2_I_9_ shown in inset; Reproduced with permission.^35^ Copyright 2016, Springer. f) Crystal structure of (CH_3_NH_3_)_3_Sb_2_I_9_; g) Comparison of the absorption coefficient of various Bi‐based perovskites and (CH_3_NH_3_)_3_Sb_2_I_9_ determined by PDS measurements; h) *J*–*V* curve of (CH_3_NH_3_)_3_Sb_2_I_9_ solar cell measured with “up” and “down” sweep with a rate of 0.1 V s^−1^. Reproduced with permission.^117^ Copyright 2016, ACS.

*Cs_3_Sb_2_I_9_*: Depending on the synthesis conditions, Cs_3_Sb_2_I_9_ forms completely different solid structure. From solution preparation, Cs_3_Sb_2_I_9_ preferentially forms 0D structure with isolated dimers of face sharing octahedrons (space group *P*6_3_/*mmc*, no. 194) similar to that of Cs_3_Bi_2_I_9_. While from solid‐state or gas‐phase reactions, it forms 2D layered structure ($P\bar{3}$ *m*1, no. 164) (**Figure** **21**a,b). In 2015, Saparov et al.^119^ reported preparation and characterization of Cs_3_Sb_2_I_9_ thin films, and the first solar cell using Cs_3_Sb_2_I_9_ as light absorbers. The prepared Cs_3_Sb_2_I_9_ derivative from two‐step deposition approach has a layered structure and shows large grains above 1 µm. The layered Cs_3_Sb_2_I_9_ film shows red color as opposed to the orange color of the 0D Cs_3_Sb_2_I_9_. The film has a bandgap of 2.05 eV, high absorption coefficients up to 10^5^ cm^−1^, an ionization energy of 5.6 eV, and better stability in ambient air than MAPbI_3_ films (Figure 21c). Unfortunately, the PV device with an architecture of glass/FTO/c‐TiO_2_/Cs_3_Sb_2_I_9_/PTAA/Au showed PCE below 1% and a low open‐circuit voltage between 0.25 and 0.3 V. They ascribed the low performance of Cs_3_Sb_2_I_9_ to the deep defects that promote nonradiative recombination, which is substantially suppressed in MAPbI_3_ with shallow defects. Later, 0 D structure was also synthetized to fabricate solar cell devices and displays champion efficiency of 0.84% with improved Voc of 0.60 V.^118^

*Rb_3_Sb_2_I_9_*: As mentioned above, Cs_3_Sb_2_I_9_ is inclined to form dimer type 0D structure when obtained via a solution process. However, in 2016, Harikesh et al.^35^ found that when Cs is replaced by Rb, the resulting compound can easily form layered structures via solution processing (Figure 21d). They compared the formation energies of the dimer and layered forms of A_3_Sb_2_I_9_ with A = Cs and Rb by DFT calculations. The results showed that Rb_3_Sb_2_I_9_ had higher preference for the layered phase, which is linked to the smaller ionic radius of Rb (1.72 Å), as compared to that of Cs (1.88 Å). Moreover, Rb_3_Sb_2_I_9_ is thermally stable up to 250 °C, and no phase transition between −40 to 200 °C, which is beneficial for operating in a solar cell. Additionally, they obtained a near ideal stoichiometry Rb_3_Sb_2_I_9_ film with a single 3+ oxidation state of Sb by an excess SbI_3_ treatment method. The perovskite films with the SbI_3_ treatment showed better coverage compared to the pristine films, with an absorption coefficient of >1 × 10^5^ cm^−1^ and an indirect bandgap of 2.1 eV. The solar cells based on Rb_3_Sb_2_I_9_ with poly‐TPD as HTM exhibited *J*_SC_ = 2.11 mA cm^−2^, *V*_OC_ = 0.55 V and a PCE of 0.66% (Figure 21e).

*MA_3_Sb_2_I_9_*: Unlike inorganic Rb_3_Sb_2_I_9_ and Cs_3_Sb_2_I_9_, which tend to form two different structures (layered or dimer) depending on the crystallization conditions, when organic cation MA^+^ is used, the hybrid antimony‐based perovskites MA_3_Sb_2_I_9_ only forms 0D dimer structure, with octahedral anionic metal halide units (Sb_2_I_9_)^3−^ surrounded by (MA)^+^ cations^210^ (Figure 20f). MA_3_Sb_2_I_9_ used in the photovoltaic application was first reported by Hebig et al.^117^ They prepared flat and homogeneous thin films of MA_3_Sb_2_I_9_ by a two‐step spin‐coating process followed by toluene treatment. The MA_3_Sb_2_I_9_ thin film shows a peak absorption coefficient (α) above 10^5^ cm^−1^ and an optical bandgap of 2.14 eV. Additionally, they found that the Sb‐perovskite showed no exciton peak in its absorption spectrum contrasting with the related Bi compound. The Urbach tail energy of this amorphous compound is close to 62 meV, indicating a high degree of energetic disorder, which will bring additional sources of nonradiative recombination engendering low open‐circuit voltages.^211^, ^212^, ^213^ They fabricated a planar heterojunction solar cell with the architecture of ITO/PEDOT: PSS/absorber/PC_61_BM/ZnO‐NP/Al, which showed a PCE of η ≈ 0.5%, *V*_OC_ of 0.89 V, and extremely low photocurrent densities (Figure 20h). Very recently, Boopathi et al.^118^ reported one‐step prepared MA_3_Sb_2_I_9_ through optimization of the precursor solution and HI concentrations. The highly crystalline MA_3_Sb_2_I_9_ film along with improved surface morphology contributed to the record PCE of 2.04% based on a planar architecture devices.

*(NH_4_)_3_Sb_2_I_x_Br_9−x_*: An even larger A cation was used by Zuo and Ding to synthesize a family of layered perovskite type light absorbers (NH_4_)_3_Sb_2_I*_x_*Br_9−_ *_x_* (0 <*x* < 9).^116^ These materials show good solubility in ethanol, variable absorption onset from 558 to 453 nm and high carrier mobility (hole of 4.8 cm^2^ V^−1^ s^−1^ and electron of 12.3 cm^2^ V^−1^ s^−1^). (NH_4_)_3_Sb_2_I_9_ solar cells gave an extraordinarily high *V*_OC_ of 1.03 V and a PCE of 0.51%.

*Cs_4_CuSb_2_Cl_12_*: Aiming for high‐performance lead‐free metal‐halide perovskite, Vargas et al.^121^ incorporated Cu^2+^ into α‐Cs_3_Sb_2_Cl_9_ and yielded layered perovskite Cs_4_CuSb_2_Cl_12_, which has a direct bandgap of 1.0 eV and better conductivity than that of MAPbI_3_. Additionally, Cs_4_CuSb_2_Cl_12_ displayed high thermal‐stability, photostability, and resistance to humidity. These properties show that Cs_4_CuSb_2_Cl_12_ is a promising material for photovoltaic applications.

#### 2.2.3 Summary

Aforementioned Bi^3+^ and Sb^3+^‐based perovskites and related absorbers represent the efforts of the scientist to find alternative lead‐free active materials in PSCs. Bi^3+^‐based compounds are much less toxic, even than divalent Sn^2+^ and Ge^2+^ ions, while displaying admirable air stability among all metal‐halide hybrid absorbers. However, the trivalence oxidation state of Bi makes the related compounds usually form low dimensional phases, with large or indirect band gap (≈2 eV), high exciton binding energy (70–300 meV),^88^ and relatively low charge transport ability. On account of the disadvantages, the highest PCE of Bi‐based PSCs reported so far is only 2.1%. Though the heterovalent substitution with monovalent metal could yield a 3D double perovskites, they usually display wide bandgap or need to involve toxic elements. Actually, there is no real application of these 3D double perovskites in solar cells to date. A recent theoretical study indicates that these structural 3D double perovskites do not have a 3D electronic structure.^203^ Another problem is the poor morphology of Bi‐based perovskite, which might originate from the preferred tendency to form regular hexagonal crystalline phase. Thus, two main strategies may be used by the community to construct ideal high‐efficiency Bi‐based PSCs: (i) compositionally engineered bismuth perovskite with 3D electronic structure and therefore low bandgap and (ii) new film fabrication methods which are suitable for Bi‐based perovskite. At the same time, the development of Sb^3+^‐based perovskites is still in its infancy. Owing to the trivalence oxidation state similar with Bi^3+^, Sb^3+^‐based perovskites also have wide bandgap and low dimensional structure with low efficiency. Sb^3+^‐based perovskite has deep level defects (versus shallow levels in MAPbI_3_ 214), which is extremely detrimental to solar cells performance. Besides, Sb^3+^‐based perovskites (Cs_3_Sb_2_I_9_, Rb_3_Sb_2_I_9_) are prone to form in 0D dimer structure with poor charge transport when they are prepared via a solution process, which is also disadvantageous to high‐efficiency solar cells. Though these research results on the photovoltaic performance are unsatisfactory, they paved the way to lead‐free halide hybrid absorbers for photovoltaic applications. The dimensionality variation of bismuth and antimony‐based absorbers is shown in **Table** **2**.

**Table 2:** Dimensionality variation of bismuth and antimony‐based absorbers

B Cation	0D	1D	2D	3D
Bismuth	Cs_3_Bi_2_I_9_ 88 MA_3_Bi_2_I_9_ 88 FA_3_Bi_2_I_9_ 95 (C_3_H_5_N_2_)_3_Bi_2_I_9_ 96 (C_6_H_14_N)_3_Bi_2_I_9_ 97	MA_3_Bi_2_Cl_9_ 98 (H_3_NC_6_H_12_NH_3_)BiI_5_ 100 C_5_H_6_NBiI_4_ 99 C_6_H_8_NBiI_4_ 99 (TMP)BiX_5_ (X = Cl, Br, I)^101^	K_3_Bi_2_I_9_ 36 Rb_3_Bi_2_I_9_ 36 Cs_3_Bi_2_Br_9_ 107 CsBi_3_I_10_ 100 (NH_4_)_3_Bi_2_I_9_ 103 MA_3_Bi_2_Br_9_ 102 (TMP)_1.5_Bi_2_I_7_Cl_2_ 101	Cs_2_AgBiCl_6_ 108 Cs_2_AgBiBr_6_ 38 Cs_2_(Ag_1−_ *_a_*Bi_1−_ *_b_*)Tl*_x_*Br_6_ 111 Cs_2_Ag(Bi_0.625_Sb_0.375_)Br_6_ 112 Cs_2_InAgCl_6_ (non‐Bi)^113^ MA_2_TlBiBr_6_ 105 MA_2_TlBiI_6_ 175 MA_2_KBiCl_6_ 104 MA_2_AgBiBr_6_ 104 AgBi_2_I_7_ 114
Antimony	Cs_3_Sb_2_I_9_ 119 Rb_3_Sb_2_I_9_ 35 MA_3_Sb_2_I_9_ 117	[CH_3_SC(NH_2_)_2_]_2_SbA_5_ 120	Cs_3_Sb_2_I_9_ a) 119 Rb_3_Sb_2_I_9_ a) 35 (NH_4_)_3_Sb_2_I*_x_*Br_9−_ *_x_* 116 Cs_4_CuSb_2_Cl_12_ 121	

^a)^ Transformed.

### 2.3 Copper‐Based Perovskites

**Figure 22:**
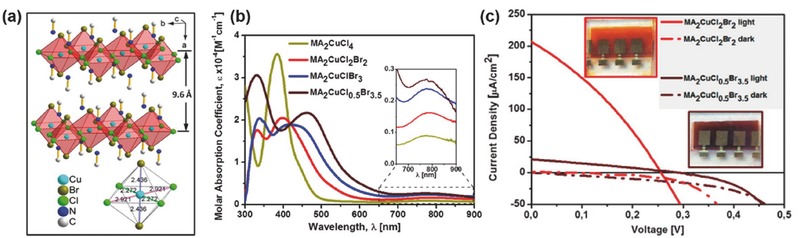
a) Crystal structure of MA_2_CuCl_2_Br_2_; b) Absorption coefficient for perovskites of the series MA_2_CuCl*_x_*Br_4−_ *_x_* showing strong CT bands below 650 nm and broad d–d transitions between 700 and 900 nm (inset); c) *J*–*V* curve of solar cells sensitized with MA_2_CuCl_2_Br_2_ (red) and MA_2_CuCl_0.5_Br_3.5_ (brown) under 1 sun of light illumination. Reproduced with permission.^39^ Copyright 2016, ACS.

Copper (Cu), one of the first‐row transition metals, is essential in the human body as part of enzymes, and also could be applied in medical research and clinical practice for radiotherapy of cancer cell.^215^ Hence Cu is relatively environmentally friendly, which is also used as a plain conductor in our daily life. Unlike conventional Pb‐based perovskites with 3D structure, Cu‐based perovskites usually form 2D layered structure, due to its smaller ionic radii. Their general formula is (RNH_3_)_2_CuX_4_, where R‐NH_3_^+^ is aliphatic or aromatic ammonium cation and X is a halogen.^122^ Cu^2+^ with an electronic configuration of 3d^9^ (t_2g_^6^ e_g_^3^), is more stable in the air than other two divalent Sn^2+^ and Ge^2+^ (**Figure** **22**a). In 2015, Cui et al.^122^ synthesized two cupric bromide hybrid perovskites, (*p*–F–C_6_H_5_C_2_H_4_–NH_3_)_2_CuBr_4_, and (CH_3_(CH_2_)_3_NH_3_)_2_CuBr_4_, with absorption from 300 to 750 nm and studied their photovoltaic performance. This is the first report on Cu‐based PSCs and showed PCEs of 0.51% and PCE of 0.63%, respectively. Both devices exhibited good air stability with less than 5% decrease of the efficiencies after 1 d in the air with humidity of 50% without encapsulation. Later, Cortecchia et al.^39^ reported Cl‐doped MA_2_CuBr_4_ perovskites, and found that the Cl was essential for stabilizing MA_2_CuCl*_x_*Br_4−_ *_x_* perovskites against copper reduction and enhancing the perovskite crystallization. By tuning the Br/Cl ratio, the optical absorption can be adjusted and extended to the near‐infrared. Further optimizing the infiltration of mesoporous TiO_2_ by 2D copper perovskites yielded a PCE of 0.017% using MA_2_CuCl_2_Br_2_ as the light harvester (Figure 22b). Moreover, Li et al.^123^ claimed the syntheses of a highly stable C_6_H_4_NH_2_CuBr_2_I compound by equimolar reaction of hydrophobic C_6_H_4_NH_2_I (2‐indoaniline) with low‐toxic CuBr_2_. The XRD patterns of the C_6_H_4_NH_2_CuBr_2_I thin film showed almost no change after 4 h of immersion in water, and the printable mesoscopic solar cell based on carbon back‐contact achieved the best PCE of 0.46% (Figure 22c). The low device performance is due to low absorption coefficient (<10^5^ cm^−1^),^39^ anisotropic charge transport in low‐dimensional structure and heavy mass of the holes. Moreover, the existence of Cu^2+^ reduction could introduce higher trap density, which is unfavorable to photovoltaic performance.

## 3 Conclusion and Outlook

**Figure 23:**
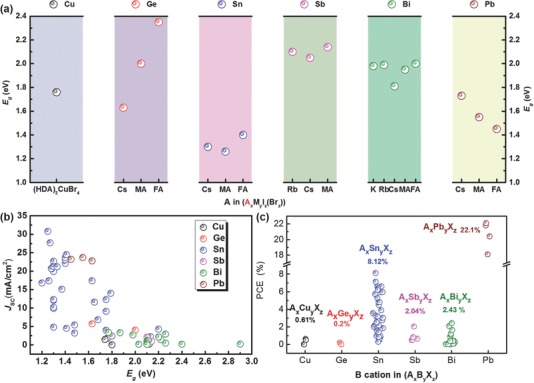
a) *E*_g_ versus the type of metal cation; b) *J*_SC_ versus *E*_g_ of the absorbers with different metal cation; c) Comparison of the performance of the device between different lead‐free absorbers (data were analyzed based on Table 1).

We have thoroughly reviewed a series of lead‐free halide hybrid absorbers with various metallic cations including Sn, Ge, Bi, Sb, and Cu etc. in the context of solar cells application. It has been proved that the variation of halide elements in the X position of a typical perovskite material will change the *E*_g_ significantly and the trend follows the electronic negativity of the halide ions.^3^ Similarly, the variation of A cations also influences the *E*_g_ in various ways depending on the type of central metal ion^3^ (**Figure** **23**a). In the case of lead halide perovskite, the *E*_g_ is decreasing with the increase of radii of A cations. However, in the case of Ge halide perovskite, the trend is reversed with CsGeI_3_ has the smallest *E*_g_. For Sb and Bi‐based absorbers, the lowest *E*_g_ appears when Cs^+^ is used as the A cation. The *E*_g_ will increase no matter the radii of A cation are larger or smaller than Cs^+^. This phenomenon may be due to the change in the dimensionality of the material. In the case of Sn‐based material, MASnI_3_ has the lowest *E*_g_. Among all the metal cations under study, Sn perovskite has the lowest *E*_g_ 132 while Ge perovskite gives the highest *E*_g_.^95^ The differences in *E*_g_ reflect directly in the short current density (*J*_SC_) of the corresponding devices. As can be seen from Figure 22b, Sn‐based perovskites provide the highest *J*_SC_, while materials based on Bi and Sb yield the lowest *J*_SC_ due to the low dimensionality and wide *E*_g_. A comparison of the device performance between different lead‐free absorbers is visualized in Figure 23c. It is clear that there is still a huge gap in PCEs between them and Pb‐based perovskite. Hence, a wise decision should be made while efforts should be put in the most plausible direction.

As the most studied lead‐free perovskites, Sn‐based absorbers with the retaining 3D framework like Pb analogs hold excellent optoelectronic properties, especially the narrow bandgaps and high carrier mobilities. However, the notorious “self‐doping” effect impedes their further development. To suppress “self‐doping” effect, various tin halide additives and organic reducing agents were introduced into the active layers. As usual, the bad morphology of perovskite films is detrimental to the device performance. Thus, strategies containing the use of additives, solvent engineering, vacuum process, vapor‐assisted solution process (VASP), and thermal annealing were adopted to improve the quality of tin perovskites film. Recently, great progress was made in an inverted device architecture. The combination of SnF_2_ additive and antisolvent treatment with chlorobenzene gave a new record efficiency of 8.12% with good reproducibility from (FA)_0.75_(MA)_0.25_SnI_3_‐based PSCs. More importantly, 80% of PCE retained after 400 h. Among the various types of tin‐based perovskites (different A cations), different cations showed different advantages. First, Sn‐based perovskites with FA cation showed higher efficiency and stability than MA and Cs counterparts. It has been speculated that FA cation can endow the perovskite with higher formation energies of Sn vacancies^139^ and more resistance against oxidation.^133^, ^138^ On the other hand, FA‐based hybrid perovskites have better solubility than all‐inorganic perovskites. According to the summary in Table 1, no spin coating with solvent‐engineering was used in Cs‐based perovskites due to their poor solubility, but in the case of FA counterparts, most good results^28^, ^56^, ^57^ originated from solvent‐engineering resulted in excellent morphology. Second, mixing organic cations at A position seems an effective method to improve devices performance. FA–MA mixed tin perovskite showed *V*_OC_ up to 0.61 eV, while FA(MA)–BA(PEA) mixed Sn perovskite yielded a 2D structure with improved air stability. Thirdly, Cs‐based perovskites have the best thermal stability up to 200 °C. Due to the high valence Sn^4+^ cation, Cs_2_SnI_6−_ *_x_*Br*_x_* showed the highest environment stability, the PSCs based on which were processed in the air without using any additives.

Despite that the Sn‐based absorbers attained a promising efficiency of ≈8%, it is still far from the best Pb‐based perovskites. “Self‐doping” effect will still be a challenge to all the researchers working on Sn‐based perovskite. A deep insight into the mechanisms of “self‐doping” effect is crucial for achieving efficiency up to 15% or higher. A more suitable procedure only employs “intermediate agent” that could be removed in the final stage or even without using any sacrificial additives. Successful compositional engineering could help to give efficient and stable compounds similar to Pb‐based perovskites. Additionally, high‐performance and dopant‐free HTMs could also assist in achieving more efficient and stable Sn‐PSCs.

For the absorbers based on metals beyond the group 14, owing to the low dimensionality and wide band gaps, the photovoltaic performance of Bi, Sb and Cu‐based devices are still unsatisfactory with efficiency ≈2%. More efforts are needed at two possible directions to open up the avenues toward high‐performance Bi‐based absorbers. The first one is new double perovskites, which may involve a lot of theoretical calculation and the corresponding experimental study. So far, researchers have used Cs_2_AgBiBr_6_ to make device with PCE up to 2.43%. The other one is the ferroelectric perovskites represented by Bi‐based compounds. As far back as 1956, photovoltaic (PV) effect has been found in oxide perovskite BaTiO_3_ which has no lead elements.^216^ Afterward, more lead‐free oxide perovskites were studied on PV effect, such as BiFeO_3_,^217^, ^218^ BiMnO_3_,^219^ [KNbO_3_]_1−_ *_x_*[BaNi_1/2_Nb_1/2_O_3−_ *_δ_*]*_x_* 220 and Bi_2_FeCrO_6_,^221^ etc. So far, the highest PCE among all oxide perovskites was obtained as 8.1% by using double‐perovskite Bi_2_FeCrO_6_.^221^ The ferroelectric oxide perovskites often showed exceptionally high photovoltages, which are normally much larger than their bandgaps.

For the transition metal Cu‐based absorbers, like 2D lead perovskites, their wide compositional tunability, and increased environmental stability are their intrinsic advantages. For example, one can introduce optoelectronically active organic cations to increase optical absorption cross‐section and improve vertical charge transport. Another approach is to make multidimensional (MD) perovskites by mixing the 2D and 3D materials.

Among all the above‐mentioned lead‐free absorbers, Sn‐based perovskites possess the most efficient PCE up to 8.12% while other absorbers showed only below 2.1%, although Bi‐based absorbers showed the highest air stability. Moreover, theoretical calculation^222^, ^223^ indicated that efficiencies above 15% could be obtained from MASnI_3_ PSCs. Thus, we argue that the Sn‐based absorbers are the most promising surrogate for Pb in PSCs. However, we have bear in mind that Sn element is still harmful to the human body in their practical utilization,^224^, ^225^ while Bi, Sb, and Cu are more environmentally friendly. Finally, we should think about our initial question: can we get the clean power output from the new hybrid absorbers without the danger of environmental contamination?^226^ The answer may need to be found in the future development of new lead‐free hybrid light harvesting materials.

During the revision of our manuscript, we noticed that there are two important Sn‐based and one bismuth‐based absorbers reported recently. Firstly, ethylenediammonium(en) cations were incorporated into FASnI_3_,^62^ MASnI_3_ and CsSnI_3_,^55^ and then form so‐called “hollow” {en}ASnI_3_ perovskite. For the sake of incorparation of appropriate amount of en cations, the 3D structure of perovskite is retained while perovskite film morphology is significantly improved. Finally, the best‐performing solar cells display a high efficiency of 6.63% of {en}MASnI_3_ and 7.14% of {en}ASnI_3_, respectively. These results are presented in table 1. Furthermore, Zhang et al. report a novel two‐step vacuum deposition procedure to get homogeneous transformation of BiI_3_ to MA_3_Bi_2_I_9_ for highly compact, pinhole‐free, large‐grained films. The solar cells realized a record PCE of 1.64% and also a high EQE approaching 60%.^227^

## Conflict of Interest

The authors declare no conflict of interest.
